# Recent progress of nanomaterials-based composite hydrogel sensors for human–machine interactions

**DOI:** 10.1186/s11671-025-04240-8

**Published:** 2025-03-29

**Authors:** Yuyang Lin, Aobin Wu, Yitao Zhang, Haiyang Duan, Pengcheng Zhu, Yanchao Mao

**Affiliations:** Key Laboratory of Materials Physics of Ministry of Education, School of Physics, Zhengzhou University, Zhengzhou, 450001 China

**Keywords:** Nanomaterials, Composite hydrogel, Wearable sensors, Human–machine interactions

## Abstract

Hydrogel-based flexible sensors have demonstrated significant advantages in the fields of flexible electronics and human–machine interactions (HMIs), including outstanding flexibility, high sensitivity, excellent conductivity, and exceptional biocompatibility, making them ideal materials for next-generation smart HMI sensors. However, traditional hydrogel sensors still face numerous challenges in terms of reliability, multifunctionality, and environmental adaptability, which limit their performance in complex application scenarios. Nanomaterial-based composite hydrogels significantly improve the mechanical properties, conductivity, and multifunctionality of hydrogels by incorporating conductive nanomaterials, thereby driving the rapid development of wearable sensors for HMIs. This review systematically summarizes the latest research progress on hydrogels based on carbon nanomaterials, metal nanomaterials, and two-dimensional MXene nanomaterials, and provides a comprehensive analysis of their sensing mechanisms in HMI, including triboelectric nanogenerator mechanism, stress-resistance response mechanism, and electrophysiological acquisition mechanism. The review further explores the applications of composite hydrogel-based sensors in personal electronic device control, virtual reality/augmented reality (VR/AR) game interaction, and robotic control. Finally, the current technical status and future development directions of nanomaterial composite hydrogel sensors are summarized. We hope that this review will provide valuable insights and inspiration for the future design of nanocomposite hydrogel-based flexible sensors in HMI applications.

## Introduction

With the development of wearable human–machine interaction technologies such as wearable watches, flexible screens, and virtual reality (VR), stretchable sensor devices have received extensive attention and possess a significant demand [1–3]. From smart electronic devices and virtual reality to robotic arm control, human–machine interactions (HMIs) have become an indispensable component in our daily lives and industrial production. It not only significantly enhances the collaborative capability between humans and machines, achieving efficient information transmission and command execution, but also provides extensive possibilities for interaction between humans and machines [4, 5]. However, traditional electronic devices face certain challenges in meeting the requirements of modern wearable HMIs. Firstly, traditional electronic devices are often the connection and packaging of rigid electronic chips and sensors, which have poor flexibility, severely restricting body movements and making it difficult to adapt to large-scale deformations during dynamic body movements [6, 7]. In addition, rigid traditional electronic devices may have biocompatibility issues in fields such as medical and health monitoring, direct contact of traditional electronic devices with human skin may cause skin allergic reactions or discomfort [8, 9]. Furthermore, traditional electronic devices lack self-healing capabilities and usually require replacement or repair after damage, causing certain resource wastage and environmental pollution [10]. Therefore, it is necessary to develop new human–machine interaction sensor technologies with good flexibility, biocompatibility, and self-healing capabilities to achieve a better human–machine interaction experience. Hydrogel is an extremely hydrophilic three-dimensional network structure flexible material that can rapidly swell in water without dissolving, endowing it with characteristics such as high hydrophilicity, stimulus responsiveness, and biocompatibility. In the field of flexible electronic devices, hydrogels have received extensive attention due to their flexibility, biocompatibility, and self-healing properties [11–15]. Therefore, hydrogel material has extensive application potential in wearable HMIs.

Composite hydrogels based on carbon, metal, MXene, and other nanomaterials, due to their excellent electrical conductivity, outstanding flexibility, self-healing capability, and excellent biocompatibility, are considered ideal candidate materials in the field of wearable HMI sensors [16, 17]. For example, in human–machine interaction applications of virtual reality (VR) and augmented reality (AR), carbon nanomaterial-enhanced hydrogels can be used to manufacture flexible pressure sensors and stress sensors, which can accurately detect subtle tactile and pressure changes [18]. In the field of electronic skin human–machine interaction, the composite hydrogel using nanoscale liquid metal leverages the flow characteristics of liquid metal, giving the sensor excellent conductivity and plasticity. This innovation overcomes the limitation of traditional rigid electronic devices that cannot be bent, allowing the sensor to adapt to large degrees of stretching and bending, thus maintaining performance in complex working environments [19]. In the field of smart wearable human–machine interaction, the application of MXene hydrogels offers new possibilities for the production of wearable devices such as smartwatches and health monitoring bands. By monitoring physiological signals such as heart rate and blood pressure, these devices can provide users with real-time health data [20, 21]. Composite hydrogels based on nanomaterials can develop stretchable sensors based on different mechanisms to serve human–machine interaction sensing. For instance, using the triboelectric nanogenerator (TENG) technology, self-powered sensors that do not require an external power source can be prepared. At the same time, based on the stress-resistance response mechanism of hydrogels, the realization of tactile feedback in VR and AR applications becomes possible. Composite hydrogels based on nanomaterials also possess an electrophysiological acquisition mechanism, providing a new path for the development of hydrogels for electrophysiological monitoring [22–24]. In general, composite hydrogels based on nanomaterials hold great potential in bringing revolutionary progress to the field of HMI sensors.

In this paper, we outline different types of materials for preparing nanocomposite hydrogel, including carbon nanomaterials-based hydrogels, metal nanomaterials-based hydrogels, and MXene-based composite hydrogels. Then, the main working mechanisms of nanomaterials-based hydrogels HMI sensors are summarized, including the triboelectric nanogenerator, stress-resistance response mechanism, and electrophysiological acquisition mechanism. Next, we overview the applications of nanomaterial-based composite hydrogel HMI sensors in personal electronic device control, virtual reality game, and robotic arm control. Finally, this paper comprehensively analyzes the latest developments of nanocomposite hydrogels in the field of human–machine interaction and looks forward to future trends, aiming to promote more efficient practical applications. We hope that this review will inspire researchers to engage in innovative exploration in the field of human–machine interaction sensing based on nanocomposite hydrogels.

## Properties of nanocomposite hydrogels

**Fig. 1:**
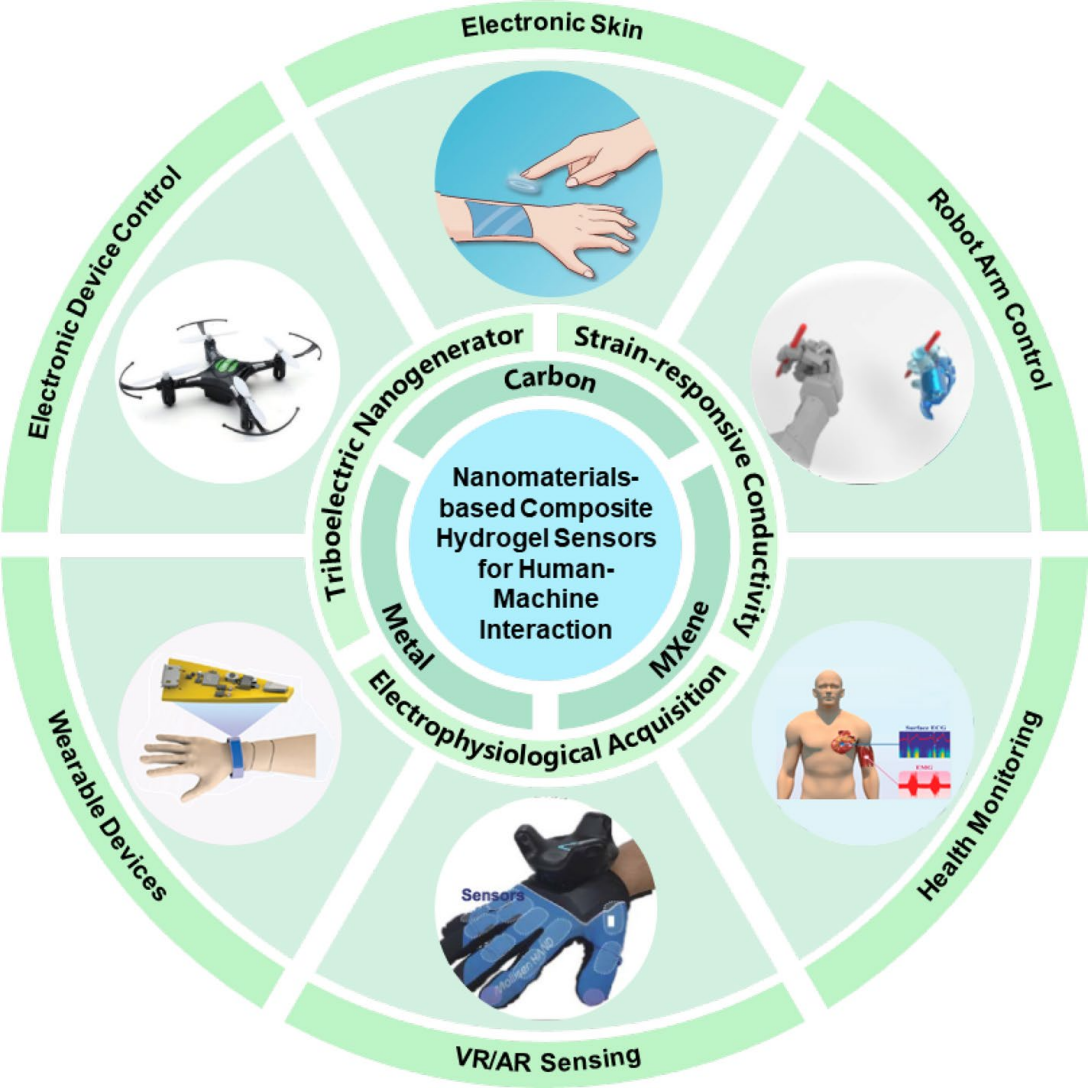
Illustration of Recent Progress of Nanomaterials-based Composite Hydrogel Sensors for Human–Machine Interaction. Image for 'Electronic Device Control': Reproduced with permission [25]. Copyright 2020, Elsevier. Image for 'Electronic Skin': Reproduced with permission [26]. Copyright 2023, Springer Nature. Image for 'Robot Arm Control': Reproduced with permission [27]. Copyright 2020, AAAS. Image for 'Health Monitoring': Reproduced with permission [28]. Copyright 2023, MDPI. Image for 'VR/AR Sensing': Reproduced with permission [29]. Copyright 2023, Springer Nature. Image for 'Wearable Devices': Reproduced with permission [30]. Copyright 2023, Wiley

**Fig. 2:**
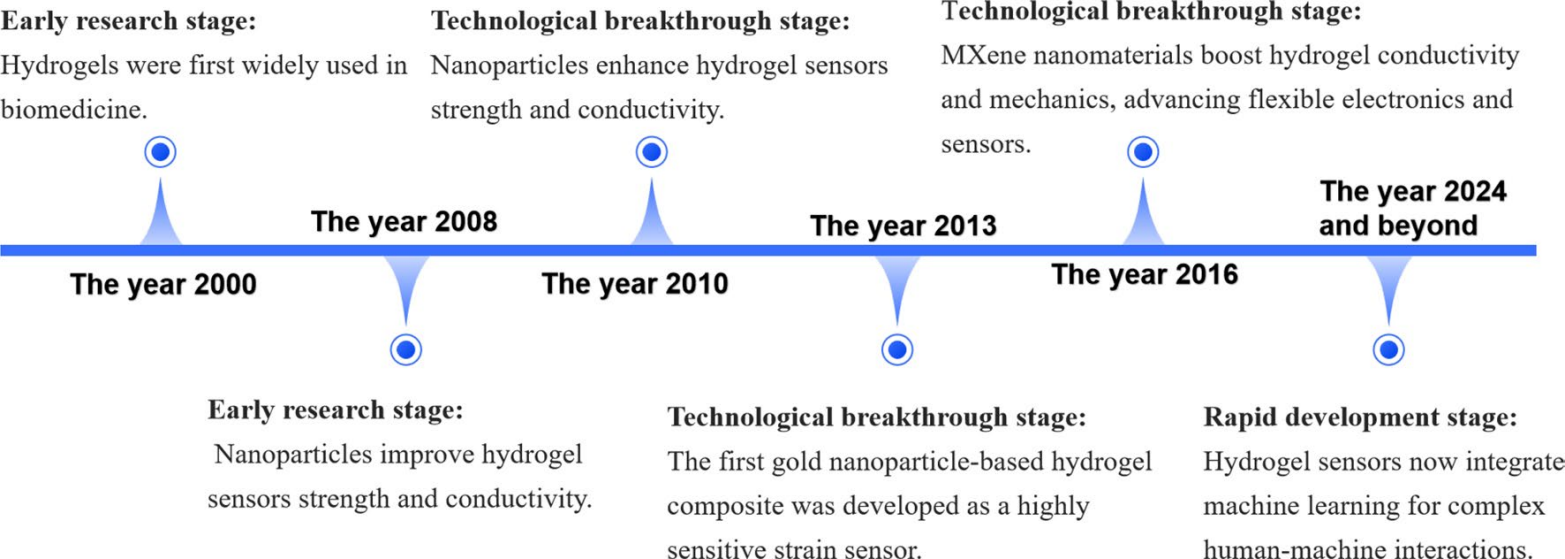
The development timeline of nanomaterials-based composite hydrogel sensors

Hydrogels are polymers made from natural or synthetic materials, known for their unique hydrophilic properties. These materials can absorb and retain a large amount of water within their three-dimensional network structures, endowing them with excellent flexibility and elasticity [13]. In physiological environments, hydrogels can effectively retain water or biological fluids, exhibiting a soft, rubbery consistency similar to living tissues, which gives them good flexibility, self-healing ability, biocompatibility, and conductivity, and thus have broad application potential [31]. With the continuous advancement in the fields of chemistry, physics, and biological sciences, coupled with the growing demands of the biomedical and pharmaceutical industries, nanocomposite hydrogels are showing new development opportunities in numerous application areas. Nanocomposite polymer hydrogels are crosslinked polymer networks that are swollen with water in the presence of nanoparticles or nanostructures. The polymers are crosslinked through chemical or physical interactions to form networks, and nanoparticles can be directly adsorbed onto the polymer chains to form double network hydrogels composed of two interpenetrating networks, significantly enhancing the flexibility of the hydrogels. Additionally, nanoparticles can also simply be trapped within the hydrogel network to increase its conductivity. Typically, the higher the concentration of nanoparticles, the stronger the conductivity [32]. These unique properties endow nanocomposite hydrogels with a broad range of application prospects, playing important roles in human–machine interfaces such as the control of small electronic devices, virtual reality game control, and machine control. Figure 1 summarizes the nanomaterials-based composite hydrogel sensors for human–machine interaction. And the development timeline of nanomaterials-based composite hydrogel sensors is presented in Fig. 2.

Since hydrogels contain a large amount of water, their performance tends to degrade over time. In order to extend and improve the water storage capacity of hydrogels, commonly used methods include designing multi-crosslinked structures to enhance their stability, adding humectants to reduce water loss, and applying surface coating techniques to prevent water evaporation. These strategies work together to effectively maintain the structure and properties of hydrogels [33]. In addition, the potential toxicity of nanomaterials is a key consideration when they are applied to the human body. Therefore, to improve the biocompatibility of hydrogels, biocompatible nanomaterials are typically selected, surface modifications can be performed to reduce interactions with human tissues, and biodegradable materials can be used to ensure that hydrogels can be safely metabolized or excreted by the body. These measures help to promote the safe clinical application of hydrogels [34]. In general, for nanoparticle-composite hydrogels, the nanoparticles are released gradually over time, whereas close-packed hydrogel structures are able to capture more nanoparticles. This is due to the higher cross-link density and smaller pore size of the close-packed structure, properties that more effectively restrict the movement of nanoparticles, making it more difficult for them to be released from the hydrogel. This ability to control the release of nanoparticles is critical to maintaining the long-term stability and functionality of the hydrogel [35]. To further limit the movement of nanoparticles, methods such as surface modification and crosslinking enhancement are often employed. Surface modification can reduce the interaction of nanoparticles with the hydrogel matrix by altering their surface properties, while cross-linking enhancement improves the overall stability of the hydrogel network by increasing the number and strength of cross-linking sites. These strategies not only effectively maintain the structure and properties of nanocomposite hydrogels, but also allow for more precise application control in areas such as drug release, tissue engineering, and biosensing [36].

### Carbon nanomaterials-based composite hydrogels

In the preparation of composite nanomaterial hydrogels for human–machine interaction (HMI), carbon nanomaterials have been widely applied due to their versatile advantages [18, 46–48]. Firstly, the incorporation of carbon nanotubes promotes both chemical and physical cross-linking of hydrogels. Physical cross-linking is mainly realized through van der Waals forces and hydrogen bonding between carbon nanotubes and polymer chains, while chemical cross-linking is through covalent bonding. This dual cross-linking mechanism can significantly improve the mechanical strength and stability of hydrogels [49–51]. Secondly, carbon nanomaterial hydrogels exhibit excellent tunability, as the type, size, morphology, and dispersity of carbon nanomaterials can be precisely controlled by selecting appropriate preparation methods and process conditions. This makes it possible to design and synthesize carbon nanomaterial hydrogels with specific functionalities and application requirements, meeting the demands of high-performance flexible electronic devices [52]. Moreover, they can enhance the stability and self-healing capabilities of hydrogels through interactions between carbon nanomaterials and polymer chains [53, 54]. Additionally, carbon nanomaterial hydrogels serve as excellent conductive media, promoting rapid electron transfer and efficient energy storage, thereby ensuring the stability and efficiency of hydrogels in the fields of energy storage electronics [54–56]. Common carbon nanomaterials-based hydrogels used for human–machine interaction include carbon nanotubes (CNTs) and graphene [57–60].

**Fig. 3:**
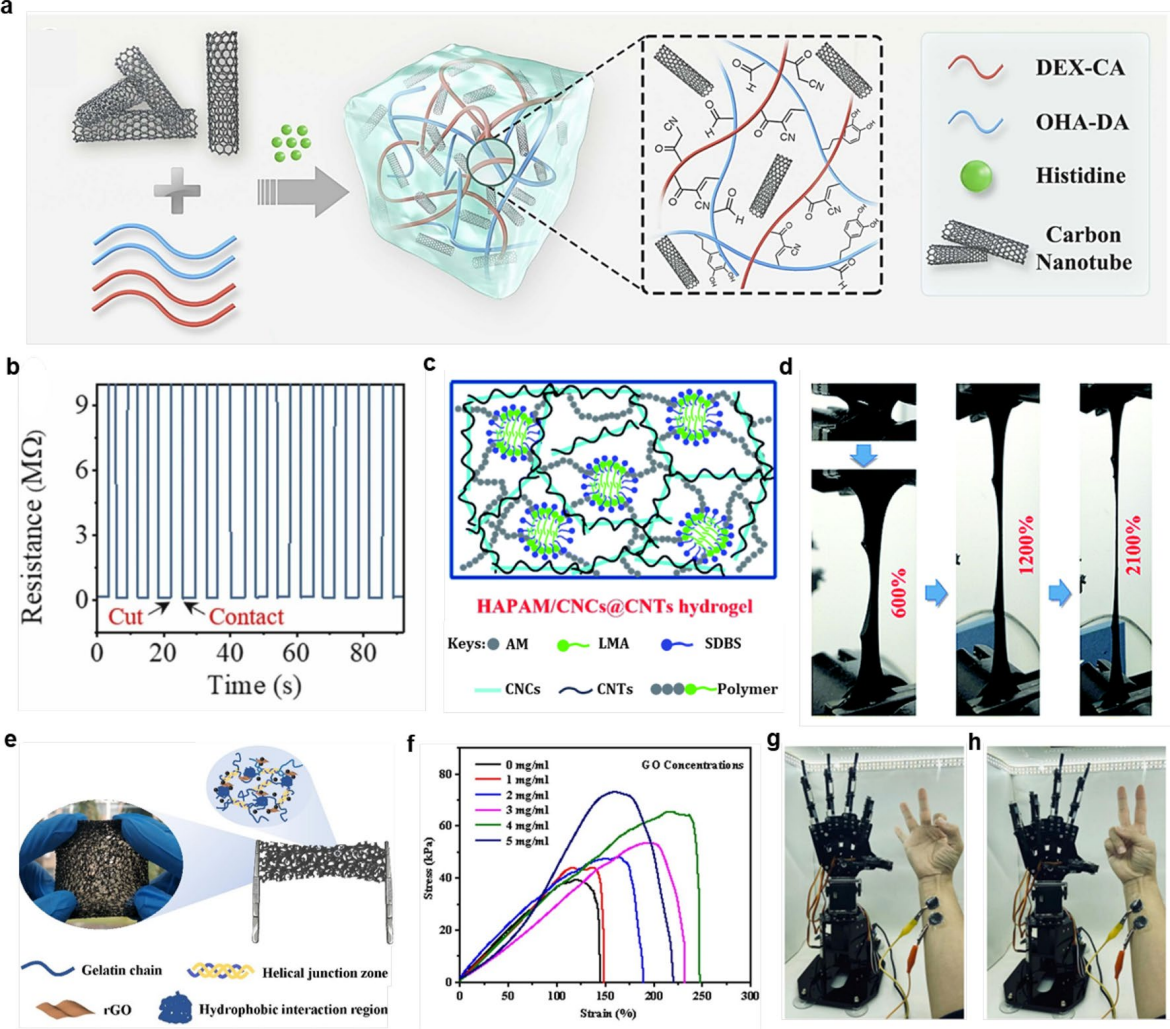
Composite hydrogels prepared from carbon-based nanomaterials. **a**, **b** Structures of multifunctional wearable electronic devices based on carbon nanotube-reinforced nanocomposite hydrogels and their resistive properties after shear and recovery [61]. Copyright 2024, Elsevier. **c**, **d** Structure and tensile properties of anti-fatigue hydrogels inspired by human tissue [62]. Copyright 2020, RSC. **e**–**h** Structure, characterization and application of gelatin/polypyrrole/reduced graphene oxide-based nanocomposite hydrogels [63]. Copyright 2022, Elsevier

CNTs, with their excellent electrical conductivity, mechanical strength, thermal stability, and unique nanostructure, are ideal choice for the preparation of carbon nanomaterial-based hydrogels. They play a key role in various application fields such as electronic devices, sensors, energy storage and conversion, and composite materials [52, 64]. Since most hydrogel-based adhesive electronic devices are difficult to remove from tissues, leading to severe electronic waste and ultimately affecting the stability and sensitivity of output signals, Ding et al. [61] designed and fabricated a novel CNT-based self-healing hydrogel with the unique ability to sense various body movements and provide on-demand removability for wearable flexible electronics. As shown in Fig. 3a, dopamine-modified oxidized hyaluronic acid (OHA-DA) is synthesized by conjugating dopamine to oxidized hyaluronic acid (OHA) using 1-Ethyl-3-(3-dimethylaminopropyl)carbodiimide/N-Hydroxysuccinimide (EDC/NHS) as a coupling agent, while cyanoacetate end-functionalized dextran (DEX-CA) is obtained from the condensation reaction between dextran and cyanoacetic acid. In the presence of CNTs, histidine is added to the mixture of OHA-DA and DEX-CA solutions, resulting in a CNT-based hydrogel with a porous network structure that achieves strong flexibility and self-healing ability in the hydrogel. Figure 3b quantitatively shows the healing ability of the hydrogel after being cut; the resistance of the hydrogel increases to infinity after being cut, but it immediately returns to its original value after the separated hydrogel blocks come into contact and self-heal. Moreover, for the hydrogel that heals after each fracture, the resistance can fully recover without any loss even after multiple fracture/self-healing cycles. This CNT-based nanocomposite hydrogel can further be used as a flexible wearable sensor. The excellent conductivity, tunable flexibility, and self-healing ability of this hydrogel enable the sensor to generate and output precise and stable electrical signals in complex application environments.

CNTs not only play a crucial role in enhancing the self-healing ability of hydrogels but also demonstrate great potential in improving the conductivity and fatigue resistance of hydrogels [65, 66]. Inspired by the structure of human tissues, Su and colleagues [62] have developed a HAPAM/CNCs@CNTs nanocomposite hydrogel based on partially crystalline fiber-like cellulose nanocrystals/carbon nanotubes (CNCs@CNTs). As shown in Fig. 3c, an acrylamide (AM) monomer solution and a small amount of hydrophobic copolymer monomer dodecyl methacrylate (LMA) are dissolved in deionized water, with the surfactant sodium dodecylbenzene sulfonate (SDBS) added to prepare the acrylamide (AM) monomer solution. In this work, CNTs are selected as the conductive element due to their fibrous shape and high conductivity. Meanwhile, amphiphilic CNCs with high crystallinity are used to disperse CNTs, obtaining a homogeneous CNCs@CNTs aqueous nanohybrid. In this structure, the long, linear CNTs are completely surrounded by rod-like CNCs, which greatly enhances the dispersion stability and forms a partially crystalline conductive network with nanoscale structure, significantly improving the conductivity of the hydrogel. Figure 3d shows that even when the sample width is cut in half, the HAPAM/CNCs@CNTs hydrogel can still be stretched to 2100%, revealing the excellent stretchability resulting from more effective energy dissipation during stretching in the HAPAM/CNCs@CNTs hydrogel. Due to its outstanding conductivity and fatigue resistance, it shows great potential for future applications in soft robotics and human–machine interaction sensing applications.

Graphene is another member of the carbon nanomaterial family, which, compared to carbon nanotubes, has a very high electrical conductivity, close to that of metals [67]. Additionally, graphene has a unique two-dimensional hexagonal plane structure of carbon atoms, endowing it with characteristics such as good transparency, high stretchability, and good flexibility. These properties give graphene-based hydrogels unique application potential in the field of signal capture [68–70]. Hou and colleagues [63] designed a nanocomposite hydrogel based on gelatin/polypyrrole/reduction graphene oxide (Gel/PPy/rGO) that exhibits excellent mechanical strength, biocompatibility, and electrochemical performance. As shown in Fig. 3e, a Gel/PPy/rGO porous hydrogel was prepared using yeast fermentation, and graphene oxide (GO) was reduced by ascorbic acid during the gelation process. The composite sol was stored at 4 °C for 30 min to form a porous hydrogel based on the triple-helix structure of gelatin polymer chains. Then, the porous hydrogel was immersed in an (NH_4_)_2_SO_4_ glycerol/water (1:1, w/w) solution for 9 h, and the Gel/PPy/rGO porous organohydrogel was obtained. Figure 3f shows that as the amount of GO increases, the breaking elongation first increases and then decreases, while the tensile strength monotonically increases. Figure 3g and h demonstrate the use of Gel/PPy/rGO porous organohydrogel electrodes to capture Electromyography (EMG) signals for controlling a robotic hand, achieving nearly 100% success rate in simulating the “OK” and “Yes” gestures. This high-fidelity signal capture capability provides important prospects for human–machine interaction scenarios where we can use gestures for real-time, high-precision control of virtual characters.

### Metal nanomaterials-based composite hydrogels

Metal nanomaterials have emerged as one of the candidate materials for the preparation of composite hydrogel sensors for human–machine interaction due to their unique advantages [71]. For the fabrication of human–machine interactive composite hydrogels, metal nanomaterials have received increasing research attention in recent years due to their self-healing capabilities, ease of processing, diverse functionalities, excellent biocompatibility, and stable mechanical properties [72–75]. Currently, the commonly used metal nanomaterials for the preparation of composite hydrogel sensors include silver nanowires (AgNW), liquid metals (LM), and gold nanoparticles(AuNP) [76–78].

**Fig. 4:**
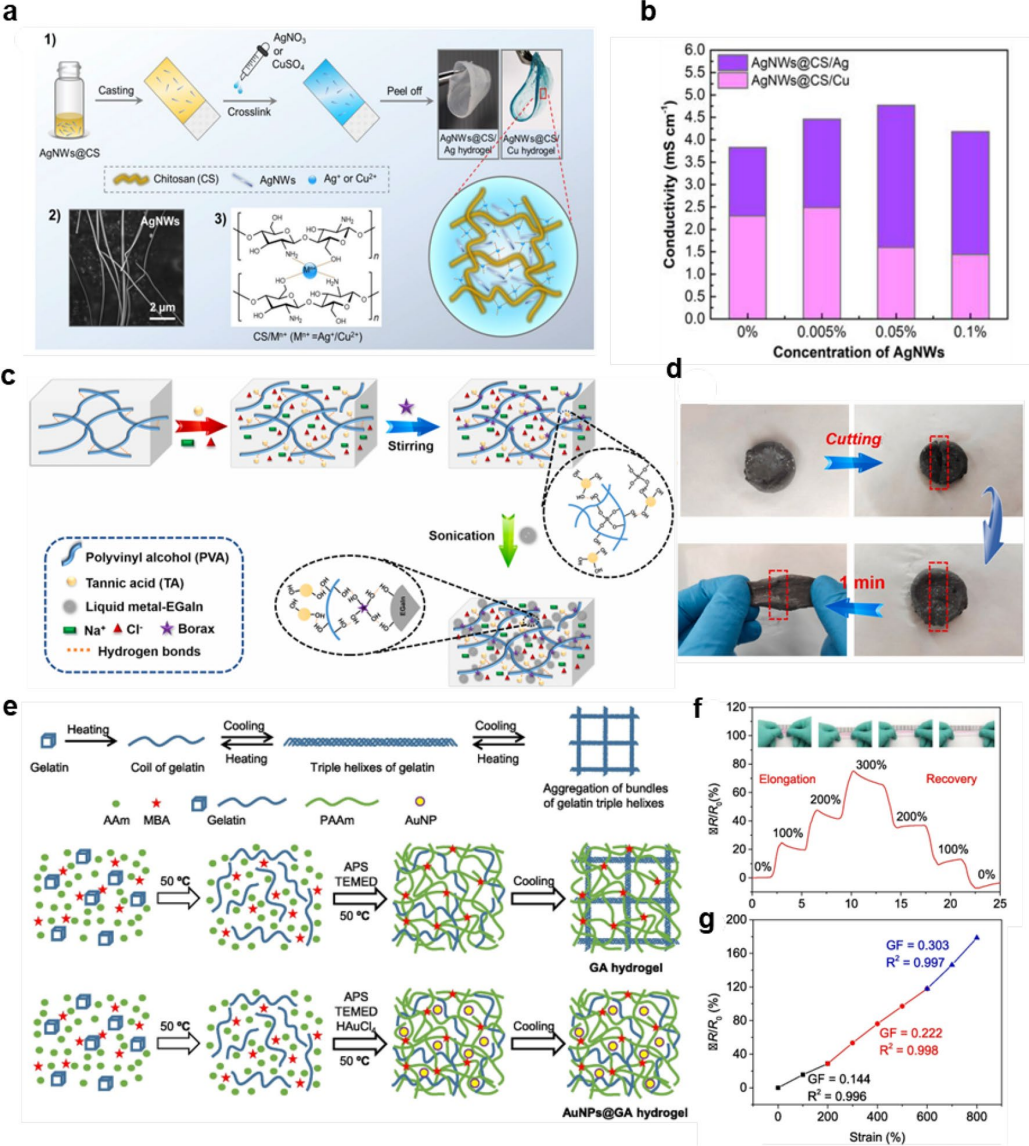
Composite hydrogels prepared from metal nanomaterials. **a**, **b** AgNW nanocomposite hydrogels for dual temperature-stress sensing, demonstrating the effect of AgNW concentration on conductivity and hydrogel output power [79]. Copyright 2019, Elsevier. **c**, **d** PVA-TA-EGaIn liquid metal nanocomposite hydrogels for self-healing and multifunctional sensing applications, including temperature and strain sensitivity [80]. Copyright 2021, Elsevier. **e**–**g** Gold nanoparticle-based composite hydrogels for strain sensing, showing the hydrogel's resistance change under mechanical deformation and its strain-resistance sensitivity in different strain ranges [81]. Copyright 2021, Elsevier

Silver nanowires are one-dimensional structural metals of silver with nanoscale diameters, and they are ideal choice for hydrogel preparation due to their nano size, good catalytic performance, antibacterial properties, and biocompatibility [82, 83]. Wang et al. [79] proposed a AgNW-enhanced composite hydrogel for temperature/stress dual sensing. Additionally, it can harvest body motion energy from the environment, making a self-powered human–machine interface sensing system possible. Figure 4a illustrates the fabrication process and structure of the AgNW-enhanced temperature/stress dual-sensing composite hydrogel. Firstly, different concentrations of silver nanowires and chitosan are mixed and cast on a glass slide, followed by the addition of AgNO_3_ or CuSO_4_ solutions. The -OH and -NH_2_ functional groups on the chitosan chains crosslink with metal ions (Ag^+^/Cu_2_^+^) through complexation, after which the substrate is peeled off to obtain an independent, flexible, and transparent hydrogel. The incorporation of silver nanowires plays a key role, not only improving the transmittance of certain composites but also enhancing the reflectivity of the hydrogel due to the silver nanoparticles’ reflective capabilities in the near-infrared light. Figure 4b shows the trend of the hybrid hydrogel's conductivity with different concentrations of AgNWs. The addition of AgNWs increases the conductivity of the AgNWs@CS/Cu hydrogel to up to 2.5 mS cm^−1^, which greatly enhances the output power of the hydrogel, suitable for constructing high-power wearable sensors.

Unlike silver nanowires which are solid at room temperature, liquid metal is a special type of metal alloy that remains in a liquid state at room temperature, known for its unique fluidity and metallic properties [84, 85]. Nanoscale liquid metal (NLM) refers to liquid metal particles or fibers with nanoscale dimensions. As a candidate material for hydrogel synthesis, NLM offers numerous advantages, including high plasticity, good electrical conductivity, and excellent biocompatibility, holding great application potential in fields such as electronics, energy, and biomedicine [86, 87]. Zhou et al. [80] used eutectic gallium indium (EGaIn) to prepare PVA-TA-EGaIn hydrogel based on liquid metal. These hydrogels possess a network structure with uniformly dispersed liquid metal, significantly enhancing the electrical conductivity and strain sensitivity of the hydrogels, making them suitable for constructing highly sensitive human–machine interaction sensors. Figure 4c illustrates the preparation process of the PVA-TA-EGaIn hydrogel. The surface of EGaIn nanoparticles has hydroxyl groups that can interact with the hydroxyl groups of PVA to maintain stability, while boric acid can form dynamic bonds with the hydroxyl groups of PVA and TA. Therefore, the hydrogel exhibits good and rapid self-healing properties. Figure 4d shows the self-healing mechanism of the PVA-TA-EGaIn hydrogel. When the hydrogel is fractured and self-healed, the hydroxyl groups between different molecular groups spontaneously crosslink to form new dynamic bonds, allowing the hydrogel to form a healing structure with strength comparable to its state before fracturing. Notably, this PVA-TA-EGaIn-based conductive hydrogel also exhibits excellent temperature sensitivity, indicating its potential as a temperature sensor. The composite hydrogel can be manufactured into multifunctional flexible wearable sensors with high temperature/strain sensitivity, conductivity, rapid self-healing, and a wide range of temperature adaptability, serving as smart biomimetic electronic skin.

In comparison to silver nanowires and liquid metals, gold nanoparticles offer controllable sizes and can have various shapes such as spherical, rod-like, star-shaped, etc., ranging in size from a few to several tens of nanometers [88, 89]. They are excellent conductive, stable, and biocompatible materials for preparing composite hydrogels, with widespread applications and numerous advantages in various fields, including catalyst and biomedicine [90, 91]. Zhang et al. [81] prepared an adhesive-tough hydrogel based on gold nanoparticles, which possesses controllable adhesion and good toughness. Figure 4e shows the preparation process and structure of the hydrogel. Firstly, gelatin is dissolved in water and then HAuCl_4_ is added. The mixture is heated to 50 °C and kept for 6 h for the reduction of HAuCl_4_ and form gold nanoparticles (AuNPs), followed by cooling to room temperature to allow the gelatin to form a triple helix structure and crosslink into a hydrogel. The presence and characteristics of AuNPs are confirmed by spectrometers and microscopes, resulting in a hydrogel with enhanced adhesion and toughness. In the AuNPs@GA (gelatin-polyacrylamide) hydrogel, the triple helix of gelatin is inhibited by AuNPs, and the gelatin chains, composed of a large number of -NH, -COOH, and -OH groups, exhibit great flexibility, thus showing greatly reduced tensile strength and significantly enhanced adhesion. The hydrogel can be further used as a sensitive strain sensor. Figure 4f shows the resistance change of the J-AuNPs@GA hydrogel during stretching. When the hydrogel is stretched or recovered by a certain proportion, its resistance also rapidly increases or decreases in accordance with that proportion, demonstrating sensitive strain-resistance characteristics. Figure 4g shows that the gauge factor (GF) of the J-AuNPs@GA hydrogel sensor is 0.144 in the low strain range (0–200%) with R^2^ = 0.996, and it increases to 0.303 in the high strain range (600–800%) with R^2^ = 0.997, exhibiting excellent strain-resistance sensitivity. The sensitive resistance changes can be used to monitor body movements, making it suitable for highly sensitive human–machine interaction sensors.

### MXene nanomaterials-based composite hydrogels

MXene as an emerging nanomaterial possesses numerous characteristics and advantages for the preparation of nanocomposite hydrogels [92–94]. This type of hydrogel combines the electrical conductivity and hydrophilicity of MXene nanomaterials with the high water absorption capacity and biocompatibility of hydrogels, forming a multifunctional, high-performance composite hydrogel material, featuring exceptional electrical conductivity, outstanding mechanical strength and flexibility, excellent hydrophilicity, remarkable environmental responsiveness, and multifunctional integration capabilities [95–97]. In addition to MXene nanosheets, two-dimensional materials also include other materials such as graphene and black phosphorus, which also have broad application potential [98, 99]. MXene nanomaterial-based hydrogels have a wide range of applications, including flexible electronic sensors, biomedical applications, and human–machine interactions [100].

**Fig. 5:**
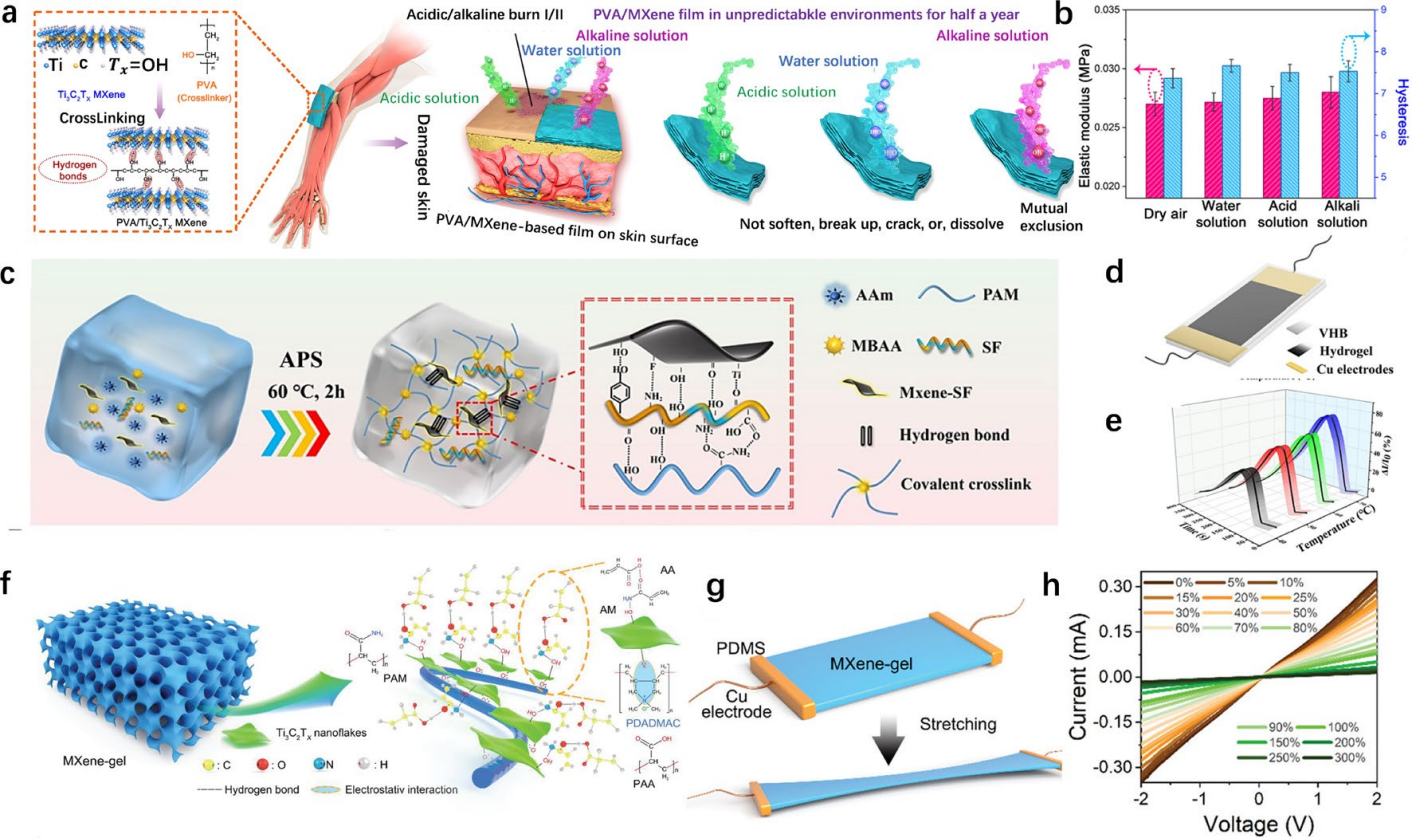
Nanocomposite hydrogels composed of MXenes. **a**, **b** Highly stable polymer cross-linked 2D MXene nanocomposite hydrogels for in vivo biomonitoring [101]. Copyright 2021, Elservier. **c**-**e** MXene nanocomposite hydrogels for surface temperature differentiation and electrophysiological signal monitoring [102]. Copyright 2024, Elsevier. **f**-**i** MXene nanocomposite hydrogels for ultra-stable strain sensors with real-time gesture recognition functionality [103]. Copyright 2023, Wiley

MXene materials, with their excellent conductivity and unique two-dimensional layered structure, achieve highly stable electronic skin functionality through synergistic integration with polyvinyl alcohol (PVA) via strong hydrogen bonding [104, 105]. In comparison, although PVA hydrogels possess good chemical stability, high mechanical strength, flexibility, and exceptional water absorption properties, they primarily serve as crosslinking agents and reinforcement matrices for MXene in this system. Zhao et al. [101] designed a highly stable electronic skin by synergistically integrating strong hydrogen bonds within MXene and polyvinyl alcohol, significantly improving the composite hydrogel's mechanical strength, chemical stability, pressure sensitivity, adsorption energy, and long-term stability in harsh environments. Figure 5a illustrates a schematic of the skin-like PVA/MXene composite film with an interactive structure within MXene nanomaterial-based hydrogels. Using PVA as a crosslinking agent, 2D Ti₃C₂Tx MXene sheets (PVA/MXene) are bonded into a layered network structure via strong hydrogen bonds, showing a hierarchically structured and chemically stable film with significant piezoelectric response and pressure sensitivity. Figure 5b shows the long-term stability test of the hybrid film-based device over 8 weeks under harsh conditions. After immersion, the pressure sensitivity of the flexible device degraded to varying degrees. After applying a constant pressure of 0.85 kPa for 8 weeks, the device showed the least degradation in dry air, with a sensitivity decrease of 8.5%. In water, pH = 3, and pH = 11 solutions, the performance decreased by 17.7%, 32.6%, and 34.7%, respectively. It can be observed that after 8 weeks of immersion, the performance of all devices remained above 65%. Specifically, after immersion in water, acidic (pH = 3), and alkaline (pH = 11) solutions, the final sensitivity was 53.3% ± 1.5%, 44.0% ± 3.6%, and 34.6% ± 4.0%, respectively. The results indicate that the flexible pressure sensors based on PVA/MXene films exhibit good stability under harsh environments. The hybrid film shows excellent pressure sensitivity under minor external stimuli and maintains good stability for over six months in water and acidic/alkaline environments. This is of significant importance for expanding the application range of flexible electronic devices in various environments.

MXene as a two-dimensional nanomaterial with excellent conductivity and mechanical properties, can also be a promising candidate for flexible electronics and sensor applications [106–108]. Li et al. [102] proposed a highly stretchable, self-adhesive, and conductive polyacrylamide/(MXene-SF) hydrogel by incorporating SF-coated MXene (MXene-SF) into a polyacrylamide (PAM) network. Figure 5c illustrates the fabrication process of the PAM/(MXene-SF) hydrogel. In the PAM/(MXene-SF) hydrogel, PAM chains are covalently crosslinked via MBAA, serving as a soft hydrogel framework. Meanwhile, multiple non-covalent reversible interactions formed between the SF functional groups on the surface of MXene nanosheets and the PAM chains, enhancing the interface bonding between MXene and the hydrogel network, endowing the PAM/(MXene-SF) hydrogel with high stretchability and strength. Figure 5d provides a schematic illustration of the structure of the PAM/(MXene-SF) hydrogel temperature sensor. The PAM/(MXene-SF) hydrogel was attached to two copper electrodes and sealed with VHB tape for thermal compensation assessment. Figure 5e shows that the current signal rapidly increased when exposed to a heat source and fully recovered when exposed to a cold source, indicating the sensor’s fast thermal response and good recovery capability. The PAM/(MXene-SF) hydrogel also exhibits high electrical conductivity (0.25 S/m) and high strain sensitivity over a large strain range, making it suitable for detecting both large-scale and subtle human movements. This study highlights the potential applications of conductive MXene hydrogel-based epidermal sensors in wearable electronics, clinical diagnostics, and health monitoring.

MXene, a class of two-dimensional transition metal carbides, nitrides, or carbonitrides, has attracted significant attention due to its excellent electrical conductivity, mechanical flexibility, and large specific surface area [109, 110]. These properties make MXene an ideal material for a wide range of applications, including flexible electronics, energy storage, environmental remediation, and biomedical devices. Moreover, the hydrophilicity of MXene and its surface functional groups enable strong interactions with polymers, facilitating the design of composite materials with enhanced properties [111–113]. Based on these advantages, Zhao et al. [103] used MXene@PDADMAC (PDADMAC refers to poly(diallyldimethylammonium chloride)) as a template, and selected common monomers such as acrylic acid (AA) and acrylamide (AM) to prepare a P(AA-co-AM)/MXene@PDADMAC semi-IPN hydrogel (hereafter referred to as MXene-gel). Based on the MXene-gel, they developed a flexible strain sensor for translating sign language into Chinese characters, which was further optimized using machine learning. Figure 5f illustrates the schematic structure of the MXene-gel, showing the orderly aggregation of PAA and PAM on the MXene@PDADMAC template. Figure 5g presents a schematic structure of the MXene-gel-based strain sensor, which uses MXene-gel as the sensing layer and copper wires as electrodes, allowing the output of the hydrogel’s electrical response to external strain. Figure 5h shows the I-V curves of the strain sensor under different strain levels (0–300%), validating that the MXene-gel-based strain sensor follows Ohm’s law, indicating its high sensitivity and fast response. Due to the intrinsic adhesiveness of MXene-gel, it can adhere to finger joint and accurately detect the bending angles of fingers. Based on this, a MXene-gel-based hydrogel senor system assisted by machine learning was developed to convert sign language into Chinese characters, promoting daily communication between hearing-impaired individuals and the outside world. This study showcases the immense potential of MXene-based hydrogel in flexible electronic devices for broad applications across human–machine interactions.

Compared with graphene-based composite hydrogels, MXene-based hydrogels have unique characteristics in preparation methods, sensing mechanisms, and applications. Graphene hydrogels, with mature preparation techniques like chemical reduction, offer high conductivity, good mechanical properties, and self-healing abilities, making them ideal for flexible electronics such as temperature and humidity sensors. MXene hydrogels form three-dimensional networks through ionic pre-intercalation and metal ion crosslinking, showing high sensitivity to deformation and stress due to their layered structure, suitable for high-sensitivity humidity sensors and human–computer interaction. CNT-based hydrogels excel in conductivity and strength for strain and pressure sensors but are costly, while metal nanomaterial hydrogels provide fast response and sensitivity for biosensors and touch panels but face oxidation issues. Overall, each type of nanomaterial varies in conductivity, sensitivity, and stability, and material selection should align with specific application needs, guided by further comparative analysis [45, 51, 114, 115]. In general, the performance comparison of composite hydrogel sensors based on nanomaterials is summarized in Table 1.

**Table 1:** Performance of composite hydrogel sensors based on nanomaterials

Filled Nanomaterial	Tensile Strain %	Breaking Strength	Electrical Conductiv-ity	GF	Adhesion Capabilities	Reference
CNTs	300	53.79 kPa	0.2 S/m	/	Y	[37]
Graphene	2009	110.97 kPa	1.69 S/m	4.01	Y	[38]
CNF	1297	870 kPa	0.32 S/m	31.51	/	[39]
AgNPs	228.5	170 kPa	3.05 S/m	6.58	Y	[40]
Egaln	2600	166 kPa	6.84 S/m	7.03	/	[41]
AuNP	1000	35 kPa	/	/	/	[42]
AuNPs	2100	/	3.41 S/m	10.23	Y	[43]
Mxene	630	625 kPa	23.65 S/m	/	Y	[44]
IL_n_ @Mxene	1903	20 kPa	1.48 S/m	7.64	Y	[45]

## Mechanisms of nanocomposite hydrogel Sensors for HMI applications

Nanocomposite hydrogels not only possess diverse characteristics and application scenarios due to their varied materials but also can integrate multiple sensing mechanisms to achieve various human–machine interaction methods. Firstly, nanocomposite hydrogels can be combined with triboelectric nanogenerators (TENG). By compounding with triboelectric materials, nanocomposite hydrogels can effectively convert external mechanical motion into electrical energy, providing a self-powering solution for wearable electronic devices and other portable electronic products. This type of composite hydrogel not only maintains the flexibility and biocompatibility of hydrogels but also enhances their output performance and stability, demonstrating great potential in the field of energy harvesting and self-powered sensors [116, 117]. Additionally, nanocomposite hydrogels can utilize the mechanism of stress-induced resistance change [118]. When nanocomposite hydrogels are subjected to external forces, the internal conductive network deforms, leading to changes in resistance. This stress sensitivity makes nanocomposite hydrogels suitable as flexible strain sensors for monitoring human movements and robotic tactile feedback systems. By adjusting the composition and structure of the composite hydrogels, precise detection and control of different stress levels can be achieved [119]. Furthermore, nanocomposite hydrogels can also utilize physiological signal acquisition mechanisms. Due to their good biocompatibility and conductivity, nanocomposite hydrogels can be directly adhered to the skin for non-invasive collection of physiological electrical signals such as electrocardiogram (ECG) and electromyogram (EMG). These hydrogel-based sensors not only provide accurate physiological data but also offer comfort during long-term wear due to their soft and breathable nature. In summary, nanocomposite hydrogels that integrate multiple sensing technologies can realize a rich array of interactive sensing modes to facilitate human–machine interaction [24, 120].

### Triboelectric nanogenerator mechanism

A triboelectric nanogenerator (TENG) is a device that can convert mechanical energy into electricity. Its working principle is mainly based on the electron transfer that occurs during the contact and separation process of two different materials, leading to a potential difference that generates an electrical signal. The electrode materials of TENG are crucial for their triboelectric activity, electrical conductivity, and mechanical flexibility. As an electrode material, hydrogels are suitable for wearable sensors due to their excellent tensile strength, deformation capacity, and biocompatibility [121–123]. By doping with conductive materials, hydrogels can effectively collect charge and convert it into electrical energy, showing great potential in applications for self-powered sensors and flexible electronic devices. Common operation modes of TENG include the vertical contact-separation (CS) mode, lateral-sliding (LS) mode, single-electrode (SE) mode, and freestanding triboelectric-layer (FT) mode [124–126]. Among these, nanocomposite hydrogels are mainly used in the CS mode, SE mode, and FT mode.

**Fig. 6:**
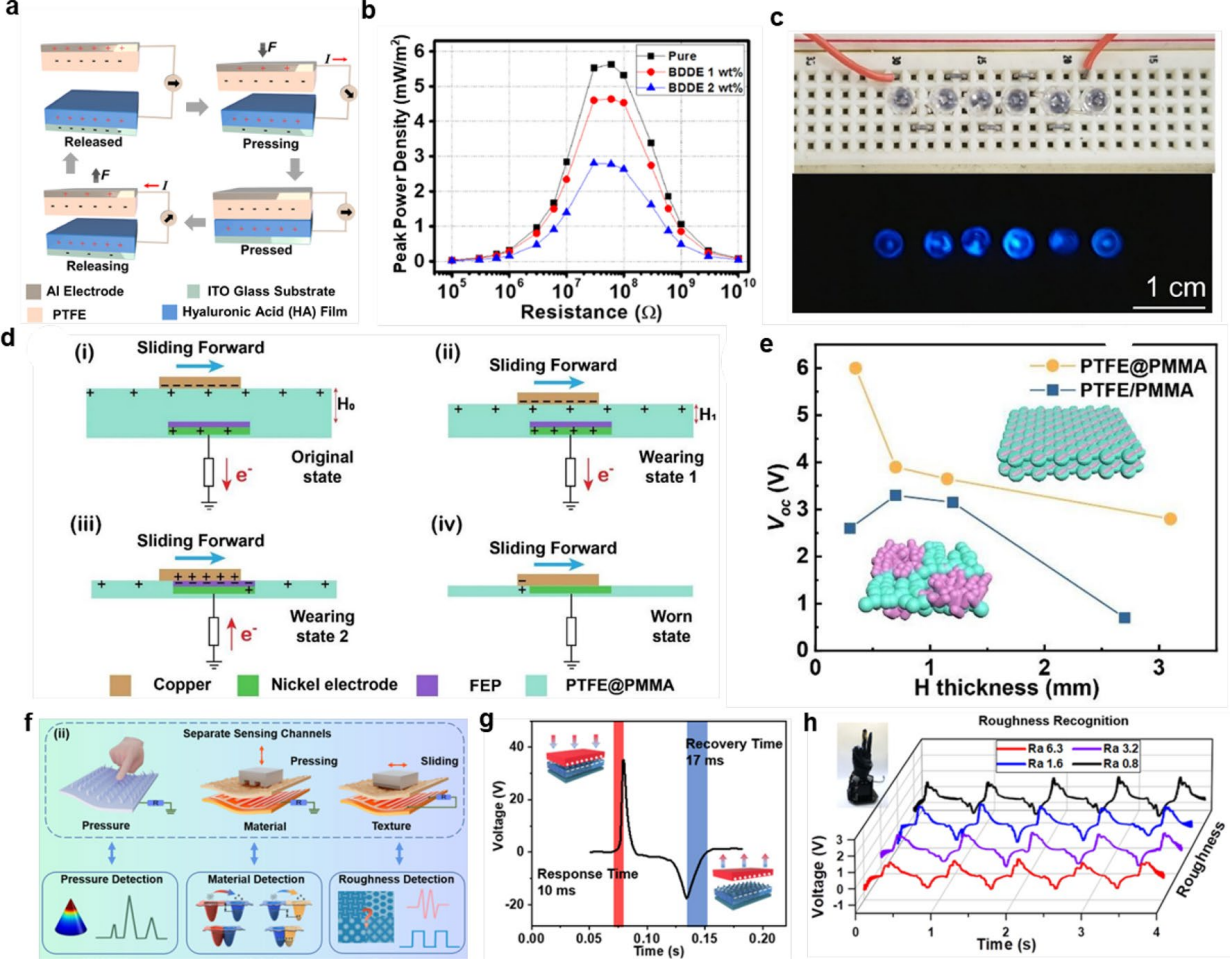
Mechanism of operation for nanomaterial-based triboelectric nanogenerators. **a**-**c** Biocompatible and biodegradable vertical contact-separation triboelectric nanogenerator based on hyaluronic acid hydrogel films [127]. Copyright 2021, Elsevier. **d**, **e** Structural principle and characteristics of a dynamic wear sensor array based on a single-electrode triboelectric nanogenerator [128]. Copyright 2020, Elsevier. **f**–**h** Schematic and working characteristics of an independent triboelectric nanogenerator for active bionic multifunctional hydrogel electronic skin based on deep learning [129]. Copyright 2023, ACS

The CS mode of TENG operates based on the contact and separation motion of two different material electrodes. In this mode, when the two different material electrodes come into contact, electrons transfer from one material to the other due to the triboelectric effect, resulting in opposite charges on the two materials. Subsequently, when the electrodes separate, a potential difference is generated between them. This potential difference can drive the flow of electrons, thereby producing a current between the two electrodes [130–132]. Kim’s groups [127] designed a hyaluronic acid (HA)-PTFE in the CS operating mode. Figure 6a illustrates the working principle of the HA-PTFE hydrogel film-based triboelectric nanogenerator. Initially, the net charge on the PTFE and HA films is minimal. When PTFE is forced into contact (pressed state) with the HA hydrogel film, electrons transfer from the surface of the HA film to the PTFE. The transferred electrons are bounded on each surface and are considered fixed in the schematic. During the release of external force (release state), the negatively charged PTFE and the positively charged HA are separated, inducing an electron flow in the external circuit. On the other hand, during the pressing process, the direction of the electron flow reverses, neutralizing the electric field on the electrodes again. Figure 6b shows that the HA-PTFE TENG has a maximum power density of 5.6 mW/m^−2^ at 60 MΩ. Figure 6c demonstrates that the output of the HA-TENG is sufficient to directly power six blue LEDs. This work proves the possibility of using hydrogels to generate and measure electrical signals with triboelectric nanogenerators, providing a platform for further application of triboelectric nanogenerators in human–machine interaction sensing in the future.

Unlike the CS mode of TENG, which requires two electrodes, the SE mode of TENG can operate with only one electrode [133, 134]. In this mode, the triboelectric layer is designed continuously and divided into two functional areas: the triboelectric effect area and the charge collection area. The triboelectric effect area is primarily responsible for contacting external objects and generating friction or mechanical deformation, while the charge collection area is used to collect the charges produced during these interaction processes [135, 136]. When friction or deformation occurs, the materials in the triboelectric effect area undergo charge transfer, generating a potential difference within the triboelectric layer. This potential difference drives the movement of charges within the layer and is eventually captured by the single electrode in the collection area. Because only one electrode is used, the charge oscillates between the triboelectric layer and ground, accumulating in the external circuit and producing alternating current. The SE mode of TENG simplifies the complexity of material selection and circuit design due to its simple structure and working principle [137]. Ren and his colleagues [128] reported an active sensor array based on TENG in the SE mode for dynamic wear monitoring and localization. Figure 6d illustratively shows the power generation principle of this TENG, where SWS consists of a copper strip, PTFE@PMMA (polytetrafluoroethylene@polymethyl methacrylate), and a nickel strip. Copper and PTFE@PMMA serve as the two triboelectric materials, and the nickel strip acts as the electrode. When the copper strip slides on the PTFE@PMMA surface, charge transfer between the contact materials produces opposite triboelectric charges on the two surfaces, thus generating electrical energy. Figure 6e indicates that when using PTFE/PMMA materials, the sensor's performance is erratic due to the non-uniform distribution of PTFE and PMMA. This non-uniformity significantly affects the surface charge density after triboelectrification, while the uniformity of PTFE@PMMA helps maintain the regularity of the open-circuit voltage (V_OC_) formed by the SWS, making it an ideal material for preparing SE mode TENG human–machine interaction hydrogel sensors.

Compared to the CS and SE modes, the FT mode of TENG stands out for its ability to operate autonomously without an external power source, typically by harvesting mechanical energy from the environment [138]. In this mode, TENG can capture energy from the environment, such as human motion, mechanical vibration, and water flow, and convert it into electrical energy to power or store energy for small electronic devices [139]. Due to its self-powering, eco-friendly, portable, and low maintenance cost, it has received considerable attention in recent years [140, 141]. Tao’s group [129] designed a bionic, ultrasensitive, and multifunctional hydrogel-based electronic skin (BHES). This hydrogel sensor incorporates an independent triboelectric nanogenerator mechanism, producing different electrical signals when sliding across different object surfaces to enable human–machine interaction. Figure 6f describes the basic operating mode of BHES, which can detect moderate normal pressure without an external power source by using micro-cone patterned hydrogel, based on the coupling of triboelectric effect and electrostatic induction. Moreover, it can identify material types through normal pressing (based on the charged contact) and recognize textures through relative sliding movement (based on the stick–slip phenomenon). Figure 6g shows the experimental dynamic response and recovery curves of BHES during pressing and releasing at approximately 2 kPa of pressure, demonstrating fast response (10 ms) and recovery (17 ms) times, which are significantly quicker than human skin (30–50 ms). Figure 6h specifically shows the application of BHES in robotic hand control, where BHES is conformally connected to the robotic hand and sequentially contacts four carbon steel surfaces with different roughness to obtain voltage signal waveforms. By using convolutional neural networks to classify and recognize the signals, a confusion matrix with an accuracy as high as 97.20% is achieved, proving its high signal sensing precision. Based on its excellent energy conversion efficiency and signal sensing precision, the independent triboelectric layer nanogenerator shows great potential for applications in wearable devices, smart sensor networks, and remote monitoring systems.

### Strain -resistance response sensing mechanism

The strain-resistance response mechanism refers to the change in conductivity of a material when it is subjected to strain deformation such as stretching or compression [142, 143]. This phenomenon has been observed in many materials, especially in certain types of polymers, nanomaterials, and composites [144]. When these materials are mechanically stressed, their internal structure and electronic properties may change, thereby affecting their conductivity [145–147]. This effect is advantageous for its high sensitivity, rapid response, ease of integration, and functional diversity, making it important in the development of smart materials and sensor technologies, such as in wearable electronic devices and flexible circuits [2, 148, 149].

**Fig. 7:**
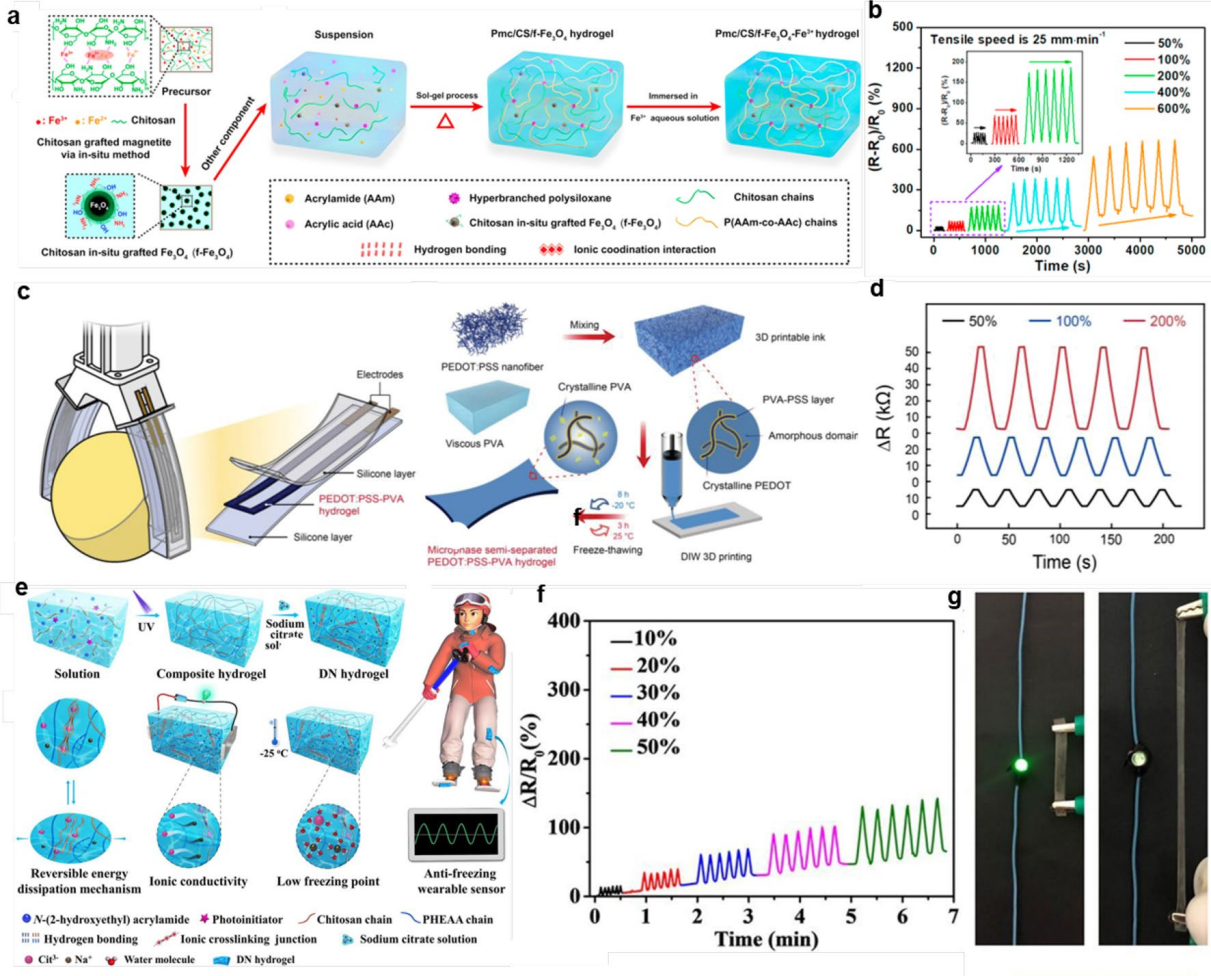
Mechanism of strain-resistant response mechanism. **a**, **b** Schematic and working characteristics of ion-covalent nanocomposite hydrogels with sensitive strain-responsive resistance [143]. Copyright 2020, Elsevier. **c**, **d** Schematic and working characteristics of highly stretchable, ultra-low hysteresis conductive polymer hydrogel strain sensors for soft robotics [150]. Copyright 2022, Wiley. **e**–**g** Schematic and working characteristics suitable for sensitive and wide-range strain and pressure sensors [151]. Copyright 2021, Elsevier

Although hydrogels possess both high mechanical performance and conductivity for wearable electronic applications, the trade-off between their strength/toughness and resistance significantly hinders their practical application in various fields. Therefore, Li et al. [143] designed a poly (acrylamide-co-acrylic acid)/chitosan/chitosan in-situ grafted magnetite (Pmc/CS/f-Fe_3_O_4_ x-Fe^3+^)ion-covalent nanocomposite hydrogel, which exhibits excellent mechanical strength and high ductility. Figure 7a illustrates the fabrication process of the nanocomposite hydrogel. First, modified magnetite nanoparticles (f-Fe_3_O_4_) are synthesized. Second, chitosan (CS), monomers, crosslinkers, and initiators are mixed to prepare the hydrogel precursor. Third, a preliminary hydrogel is formed through a sol–gel process, and ionic coordination crosslinking is performed using Fe^3+^ ions. Finally, due to the synergistic effect of nanoparticle reinforcement and various ion-covalent interactions, a uniform, ordered, and hierarchical hybrid ion-covalent network is constructed, endowing the hydrogel with superior mechanical properties and the desired conductivity. Figure 7b shows that this nanocomposite hydrogel experiences a very slow increase in the peaks and troughs of the relative resistance change curve under strains exceeding 400%, with the waveform remaining almost unchanged, indicating that the hydrogel-based strain sensor has excellent stability. In summary, the Pmc/CS/f-Fe_3_O_4_-Fe^3+^ ion-covalent nanocomposite hydrogel shows great potential for application in the field of wearable electronic devices, and its excellent mechanical properties and stability provide a possibility for expanding the practical application of hydrogels in sensors and other fields.

Despite the rapid development of strain sensors based on conductive polymer hydrogels as promising candidates for various wearable skins and soft machine sensing devices, existing strain sensors cannot fully exploit their potential when used in wearable or robotic systems due to inherent limitations such as low stretchability and large hysteresis [152, 153]. Therefore, Shen et al. [150] proposed a strain sensor based on a polystyrene sulfonate-poly(vinyl alcohol) (PSS-PVA) conductive polymer hydrogel for wearable E-skins and soft robots, as schematically depicted in Fig. 7c. By combining PSS nanofibers with polyvinyl alcohol (PVA), a unique conductive polymer hydrogel microphase semi-separated network can be created. Figure 7c also illustrates the principle and fabrication process of the PSS-PVA conductive polymer hydrogel. Through the vigorous mechanical mixing of PSS nanofibers and viscous PVA aqueous solution, followed by freeze–thaw for physical crosslinking, a microphase semi-separated hydrogel network is formed, greatly enhancing the mechanical strength and stretchability of the hydrogel without compromising its excellent electrical performance. Figure 7d demonstrates that the strain sensor based on this hydrogel can still present a regular stress-resistance response under periodic stretching at a 200% load without obvious hysteresis, exhibiting reliable response capability. It has been proven that this wearable E-skin can detect human movements, provide remote control signals for industrial robot movements, and be integrated into soft grippers for object recognition, providing a technical platform for conductive polymer hydrogels and stretchable electronic skins with enhanced sensing functions for promising smart robot systems.

Hydrogels based on the strain-resistance response mechanism not only have important applications in smart robotics but also in freeze-resistant multimodal hydrogel sensors [154–156]. To date, several highly stretchable conductive hydrogels have been developed to construct stretchable strain sensors, but flexible wearable sensors actually need to sense various deformations including elongation, compression, bending, and touch pressure. Most hydrogel sensors can only detect elongation and cannot detect a wide range of compression and pressure, which greatly limits their application scope [157, 158]. Therefore, Yang et al. [151] constructed a wearable multimodal hydrogel based on an elastic, fatigue-resistant, and freeze-resistant chitosan-polyhydroxyethyl acrylamide (CS-PHEAA) double network (DN) hydrogel. The hydrogel can detect a wide range of strain and pressure sensitively, with long-term stability and a wide working temperature range. Figure 7e illustrates the preparation process of the CS-PHEAA DN hydrogel. First, HEAA, CS, and initiator are mixed and heated to form a prepolymer solution, then crosslinked by UV irradiation to form a CS-PHEAA composite hydrogel. The hydrogels were then physically crosslinked by immersing them in saturated Na_2_SO_4_ or Na_3_Cit salt solutions to form stable DN-Sul or DN-Cit hydrogels. Finally, UV-induced crosslinking was used to form the CS-PHEAA composite hydrogel, which was then immersed in the saturated solution to construct the final physically-physically crosslinked CS-PHEAA DN-Sul or DN-Cit hydrogels. Figure 7f shows the change in relative resistance under periodic strain, which still feedbacks regular strain resistance changes even under a 400% load of periodic stretching. Figure 7g illustrates the antifreeze mechanism of the DN-Cit hydrogel, which remains soft after being placed at -20 °C for 24 h. These characteristics demonstrate their strong response reliability and provide possibilities for the practical application of wearable sensors that can operate reliably in low-temperature environments.

### Electrophysiological acquisition mechanism

The electrophysiological acquisition mechanism is based on the electrical activity of cells within a biological organism, where changes in electrical signals are measured and recorded to obtain information about the internal state of the organism [159]. Due to their excellent conductivity, mechanical flexibility, and biocompatibility, nanomaterials-based composite hydrogels have demonstrated broad application potential in the field of electrophysiological signal acquisition [160–163].

**Fig. 8:**
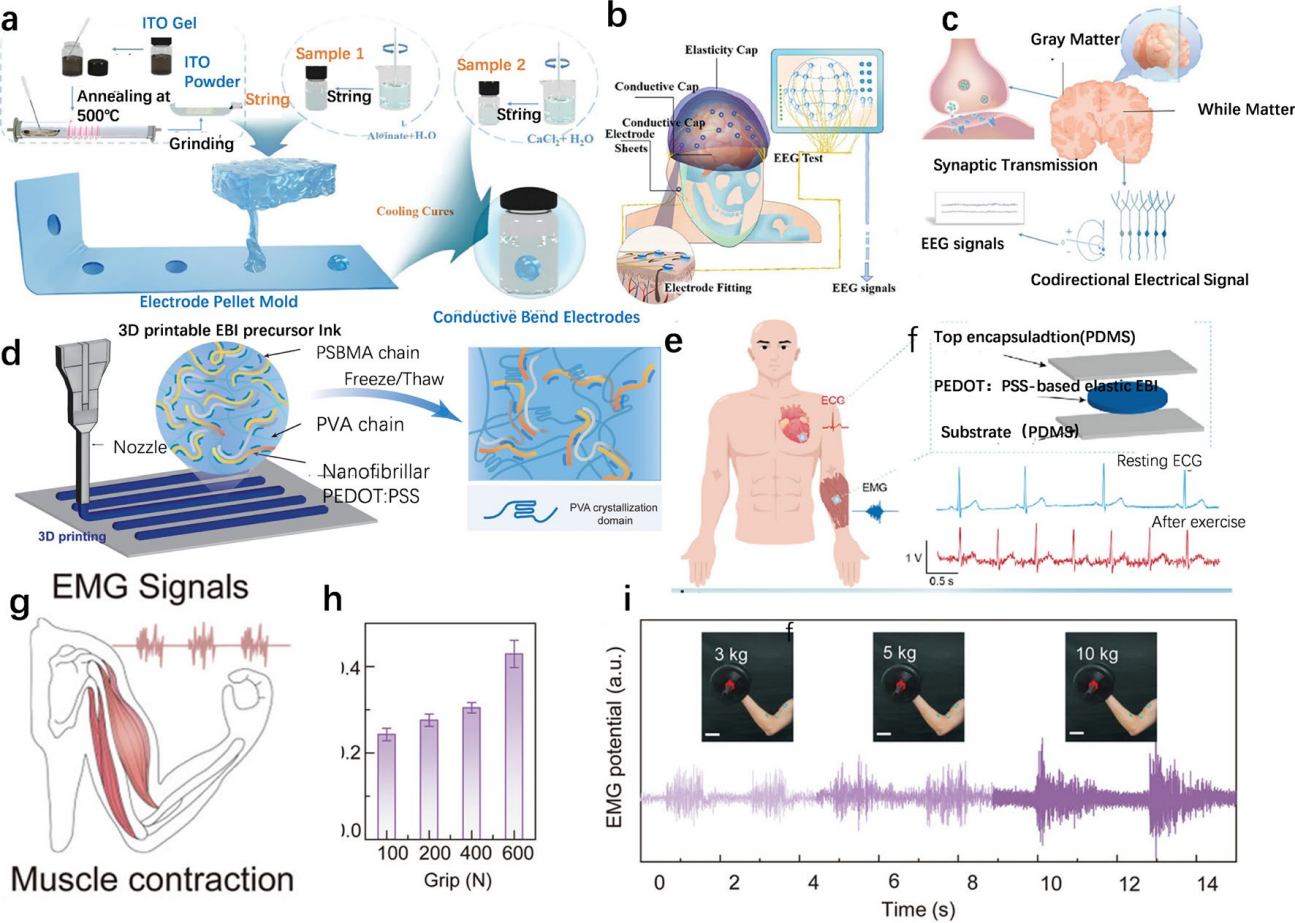
Applications and electrophysiological acquisition mechanisms of composite hydrogels. **a**-**c** Scalable elastic hydrogel interfaces and mechanisms for electrophysiological monitoring of the brain [164]. Copyright 2024, Wiley. **d**-**f** 3D-printed robust high-performance conductive hydrogel-based bioelectrical adhesion interfaces and mechanisms [165]. Copyright 2024, Wiley. **g**-**i** Thermally enhanced adhesive, antibacterial, and recyclable ionic hydrogels and mechanisms for epidermal electrophysiological monitoring [166]. Copyright 2022, Wiley

Electroencephalography (EEG) signals are records of the electrophysiological activity of neurons in the brain, reflecting the transmission of information between neurons. EEG is crucial for diagnosing neurological disorders, cognitive neuroscience research, and human–machine interaction technologies [167, 168]. Nanomaterial hydrogel electrodes have advantages and widespread applications in EEG signal acquisition, and flexible electrodes for EEG collection show great potential for human–machine interaction [169, 170]. To detect EEG signals, Hu et al. [164] have innovatively developed a non-cytotoxic spherical Indium Tin Oxide hydrogel (ITO) electrode for EEG monitoring. As shown in Fig. 8a, this electrode is prepared by embedding ITO conductive particles in a hydrogel matrix through the annealing of ITO sol, with Sn4 + replacing In3 + to enhance conductivity, and combining with sodium alginate to form a stable hydrogel. The addition of calcium salt groups improves the electrode’s adaptability to the scalp, ensuring structural stability and providing an efficient and stable solution for EEG signal acquisition. Figure 8b illustrates the operation of the conductive cap during the experiment. The elastic cap aids in conforming the hydrogel electrode to the scalp, applying pressure to the EEG cap surface. After cooling and solidification, the hydrogel EEG electrode is reheated to room temperature (20 °C) and then carefully placed onto the electrode pads of the conductive cap. Figure 8c shows the mechanism of spherical electrode measurement of EEG within the conductive cap. One fundamental principle of EEG data collection is the electric field effect. In other words, action potentials cause current flow, which in turn creates a magnetic field. This magnetic field affects the action potentials of nearby neurons, activating multiple neurons. When the dendrites of several neurons produce an electric field in the same direction simultaneously, this field, known as an open field, can be detected by EEG equipment. This study presents a method for preparing hydrogel electrodes for non-invasive EEG signal collection, addressing challenges related to conductivity and morphology. The method achieves good electrical performance, adaptable morphological characteristics, a comfortable user experience, and a rapid operational process, making it suitable for applications in human–machine interaction.

Electrocardiogram (ECG) signals are records of the heart’s electrical activity, which can be used to diagnose cardiac arrhythmias, myocardial infarction, and other heart conditions, making them essential for cardiac health monitoring and research [171, 172]. The advantages of flexible hydrogel electrodes in ECG monitoring have led to their widespread application. To enable effective detection of ECG signals, Yu et al. [165] developed a robust, high-performance, conductive Poly(3,4-ethylenedioxythiophene): Poly(styrenesulfonate) hydrogel-based electro-bioadhesive interface (EBI) using 3D printing technology. Figure 8d illustrates the 3D printing of the EBI precursor ink and its gelation mechanism through freeze–thaw processes. The composite ink demonstrated excellent 3D printability, and after printing, a single freeze–thaw cycle transformed the patterned precursor ink into high-performance Poly(3,4-ethylenedioxythiophene)-Electro-Bioadhesive Interface. The resulting PEDOT EBI exhibits excellent electrical and mechanical properties, including a high electrical conductivity of 1.2 S m^−1^, low interfacial impedance of 20 Ω, high stretchability of over 349%, toughness of 109 kJ m-3, and satisfactory adhesion to various substrates. Figure 8e shows a schematic of an elastic skin electrode based on EBI for ECG signal recording. The elastic skin electrode is designed as a three-layer structure, with the PEDOTEBI serving as the electrode and PDMS as the top and bottom encapsulation layers. Figure 8f) presents the ECG signals recorded at rest and after exercise using the fully printed EBI-based elastic skin electrode. To record ECG signals, the PDMS layer of the printed elastic skin electrode was peeled off, and the PEDOTEBI electrode was adhered to the right wrist (reference), right elbow (negative electrode), and left elbow (positive electrode), then connected with copper wires. By comparing the ECG signals before and after exercise, the elastic skin electrode effectively detected heart rate changes, demonstrating potential for monitoring conditions such as arrhythmias and other aspects of human health. Overall, this study not only offers a promising strategy to address current challenges in the advanced manufacturing of conductive polymer hydrogel-based EBI, but also provides a new pathway for the simplified fabrication of EBI-based bioelectronics and wearable devices.

Electromyography (EMG) signals are electrical signals generated by muscle contractions and can be used to assess muscle function [173]. They are crucial for diagnosing neuromuscular diseases, rehabilitation training, as well as human–machine interaction applications [174, 175]. The advantages and widespread applications of hydrogels in electrocardiogram (ECG) signal monitoring. To detect EMG signals, Liu et al. [166] proposed a skin-adhesive epidermal electrode based on a non-covalent cross-linked network of composite nanomaterial hydrogel enhanced for body temperature. Figure 8g provides a schematic of the EMG setup. The MiH hydrogel composed of polyvinyl alcohol (PVA), branched polyethyleneimine (b-PEI), and calcium chloride (CaCl_2_), exhibits strong adhesion, high conductivity, and tissue-matching modulus, making it suitable as an epidermal electrode to record EMG signals. Two MiH films serve as working electrodes on the forearm, while another film is used as a reference electrode placed on the back of the elbow. The MiH hydrogel epidermal electrode successfully collected EMG signals induced by muscle activity during arm movement. Compared to commercial Ag/AgCl electrodes (SNR = 6.4), the MiH-based electrode demonstrated lower interface resistance and higher signal-to-noise ratio (SNR = 8.5). Figure 8h shows the EMG signal strength (peak-to-peak value) under different grip forces Fig. 8i presents the EMG signals and corresponding images when lifting dumbbells of varying weights. The EMG signals indicate muscle contraction during lifting, holding, and lowering the arm. The amplitude of the EMG signals increased with the weight of the dumbbells, suggesting greater muscular effort to overcome heavier weights. In summary, as an epidermal electrode, MiH establishes a highly conformable and gap-free interface with the skin, allowing for real-time monitoring of high-fidelity EMG signals. With advancements in technology, MiH is expected to introduce intelligent functionalities and expand the development of epidermal electronic products, thereby advancing the field of medical electronics.

## Applications of nanomaterials-based composite hydrogel in HMI

Nanomaterial composite hydrogel sensors have emerged as transformative elements in human–machine interaction (HMI), enabling seamless communication between humans and devices across various applications [176, 177]. These innovative sensors offer unique advantages, such as flexibility, high sensitivity, and multifunctionality, which open up possibilities for applications in personal electronic device control, VR/AR game interaction, and robotic systems. By leveraging their exceptional material properties and advanced fabrication techniques, hydrogel-based HMI technologies are redefining user experiences and industrial standards in diverse fields.

### Personal electronic device control

Human–machine interaction composite hydrogel sensors based on nanomaterials have opened up new avenues for information exchange between humans and computers [178, 179]. They are capable of capturing and analyzing the user’s body movement signals, which may originate from bioelectric signals generated by muscle activity or from changes in the body caused by actions such as stress and deformation [180–182]. By combining nanocomposite hydrogel sensors with signal processing units and communication interfaces, the effective conversion of human dynamic signals into machine commands is achieved, thereby facilitating smooth interaction with machines [183, 184]. This interaction mode is not only intuitive and natural but also highly innovative, significantly enhancing the convenience and efficiency of human–machine interactions [185–187].

**Fig. 9:**
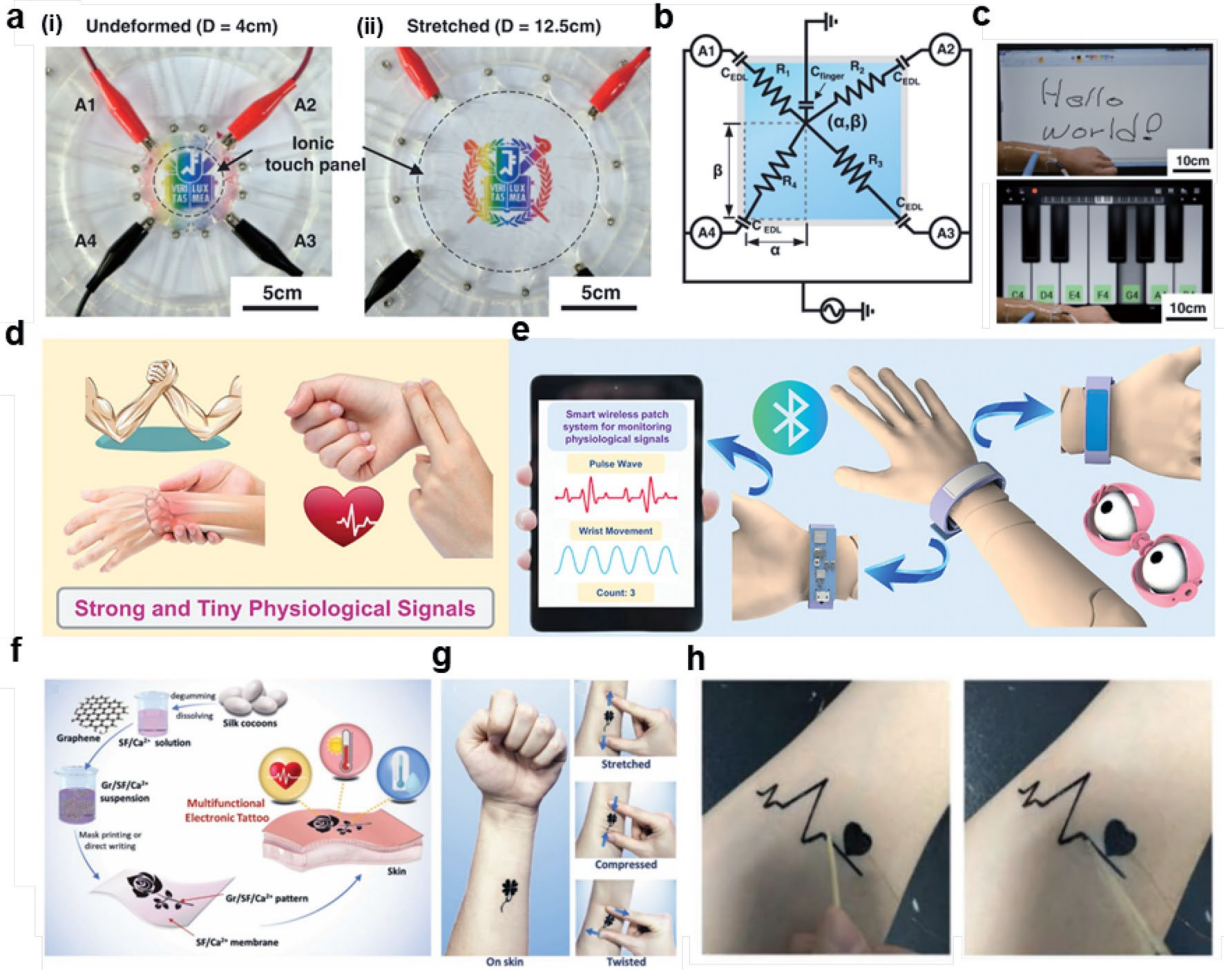
Applications of nanomaterial-based composite hydrogels for the control of small electronic devices. **a**-**c** Human–machine interface based on nanocomposite hydrogels for touch panels [188]. Copyright 2016, AAAS. **d**, **e** Nano-composite photo-curable hydrogels for wearable devices [30]. Copyright 2023, Wiley. **f**–**h** Multifunctional electronic tattoos based on graphene-based nanocomposite hydrogels [189]. Copyright 2019, Wiley

As a simple and intuitive interface for human–machine interaction, flexible touchscreens have always been a popular direction in the field of hydrogel applications. Kim et al. [188] reported a highly stretchable transparent touch panel containing lithium chloride polyacrylamide hydrogel, which is applied to flexible touch screens. Figure 9a demonstrates the hydrogel’s excellent stretchability, with (i) and (ii) showing the physical images of the scalable hydrogel touch panel whose diameter can be stretched from 4 to 12.5 cm, reaching an area of 1000% of the original size, and maintaining the same touch location detection capability under high tensile strength. Figure 9b illustrates the principle and application of the touchscreen in determining the touch position: the four corners of the touchscreen are connected to platinum electrodes and a common AC voltage is applied. When a finger touches the touchscreen, the finger changes the distribution of the electric field on the touchscreen. By analyzing the current intensity at each electrode, the two normalized distances α and β of the touch point can be calculated to determine the touch position. Therefore, this touch panel can detect various actions such as tapping, holding, dragging, and sliding. Figure 9c demonstrates application scenarios such as writing words and playing music. When the fingertip slides or clicks on the flexible touchscreen, the electric field on the surface of the touchscreen is changed by the finger. Then, the electric field signal is processed and calculated by the control circuit board, and then output to the computer. The computer receives the digital signal from the controller circuit board and interprets it as movement and click information to complete the control operation. This research further demonstrates the potential of hydrogels in flexible electronic screens for wearable human–machine interaction.

In addition to the development of flexible touchscreens utilizing hydrogel’s conductive capabilities, hydrogel’s mechanical strain sensing ability is also widely applied for wearable devices [190, 191]. The advancements in emerging wireless data collection technologies and the timely analysis of various information captured by wearable devices are increasingly gaining attention. Guo et al. [30] designed a wearable device based on a photo-curable hydrogel with excellent processing performance, high transparency, strong conductivity, and good chemical stability, utilizing a wireless strategy, which showcases a simplified structure by effectively sharing functional layers instead of the traditional two separate combinations. This device provides outstanding performance in ionic electronic sensing and electrochromic properties to quantify and visualize pressure simultaneously. Furthermore, the device combines a passive wireless system, enabling it to operate without a battery. To meet the requirements of real-time monitoring and comfortable wear, as shown in Fig. 9d, the pulse signal, a crucial indicator for blood pressure, heart issues, and vascular aging or stiffness, contains information about patterns (waveform), intensity (amplitude), and rhythm (frequency). This information can reveal the characteristics of blood flow under different states in the human cardiovascular system, and wrist motion monitoring will be beneficial for medical health diagnosis and exercise-assisted devices. Figure 9e illustrates that real-time monitoring can be achieved by integrating wearable devices with Bluetooth technology, allowing data to be sent to a custom mobile application for collection and display. When the device is in operation, the intelligent patch system first collects physiological information from the body’s surface using an embedded piezoelectric ion sensor and quantifies it as capacitive data. Subsequently, these data are converted into frequency-shift signals by an integrated detection module. The core microcontroller of the system interacts with the detection module via a specific communication interface, collects digital data, and realizes real-time data transmission through wireless technology. At the same time, the integrated chip controls the switch state of the electrochromic display based on the counting conditions of wrist motion to provide on-site direct warnings. By including information about patterns (waveform), intensity (amplitude), and rhythm (frequency), these data can reveal the characteristics of blood flow under different states of the cardiovascular system, serving as important indicators for blood pressure, heart problems, and vascular aging or stiffness. Therefore, users can monitor their pulse waves and wrist movement in real-time through the interface of a remote portable device like a tablet and count them to judge their physiological state and take appropriate actions.

Compared to sensors with single sensing modes such as flexible touchscreens, there is a rapidly growing demand for multimodal sensors capable of sensing various information such as strain, humidity, and temperature [192, 193]. Electronic tattoos (e-tattoos) are a technology that can adhere to the human skin for non-invasive, high-fidelity sensing and have become a focus of research in the field of wearable electronics. Wang et al. [189] developed a self-healing multifunctional electronic tattoo hydrogel based on a graphene/silk fibroin/Ca^2+^ (Gr/SF/Ca^2+^) composite, which can respond to various environmental changes such as strain, humidity, and temperature. Figure 9f illustrates the preparation of the electronic tattoo: SF/Ca^2+^ solution made from silk cocoons, graphene added to obtain Gr/SF/Ca^2+^ suspension, then patterned through screen printing or writing. Water spraying allows the Gr/SF/Ca^2+^ tattoo to be attached to the skin, adhering due to slight degradation. The electronic tattoo is stable, conformable, and resistant to stretching, compression, and torsion, remaining in place without misalignment, delamination, or fracturing, thus demonstrating high reliability. Figure 9g shows the conformability of this material on the skin; the clover-shaped electronic skin can closely adhere to and deform with the skin under stretching, compression, or torsion, exhibiting strong flexibility and stretchability. Figure 9h shows the self-healing electronic tattoo directly attached to the forearm for electrocardiogram measurement, with data transmission and recording performed via a Bluetooth module. The collected electrocardiogram signals are amplified and processed to extract the ECG waveform, identify characteristic peaks, and obtain parameters such as heart rate and rhythm to assess cardiac health. Thanks to the electronic tattoo as a sensing device, this measurement method has advantages such as non-invasiveness, high fidelity, portability, and long-term stability, avoiding trauma and infection risks while ensuring high-quality signals and convenient use. In summary, the electronic tattoo has broad application prospects in health monitoring, sports tracking, and human–machine interaction, promoting the development of wearable electronic devices.

### VR/AR game interaction

The exceptional properties of nanomaterial composite hydrogels present significant potential for applications in VR/AR game control [194], including but not limited to gesture recognition and control, flexible touch-sensitive devices, and human–machine interaction touchpads. These cutting-edge applications not only highlight the innovative capabilities of nanocomposite hydrogel sensors in electronic device control but also indicate that, with ongoing technological advancements, the VR/AR gaming world is poised to experience unprecedented interactive experiences and functional expansions [195, 196]. As research progresses and technology matures, we anticipate a wealth of innovative applications across various fields [197, 198].

**Fig. 10:**
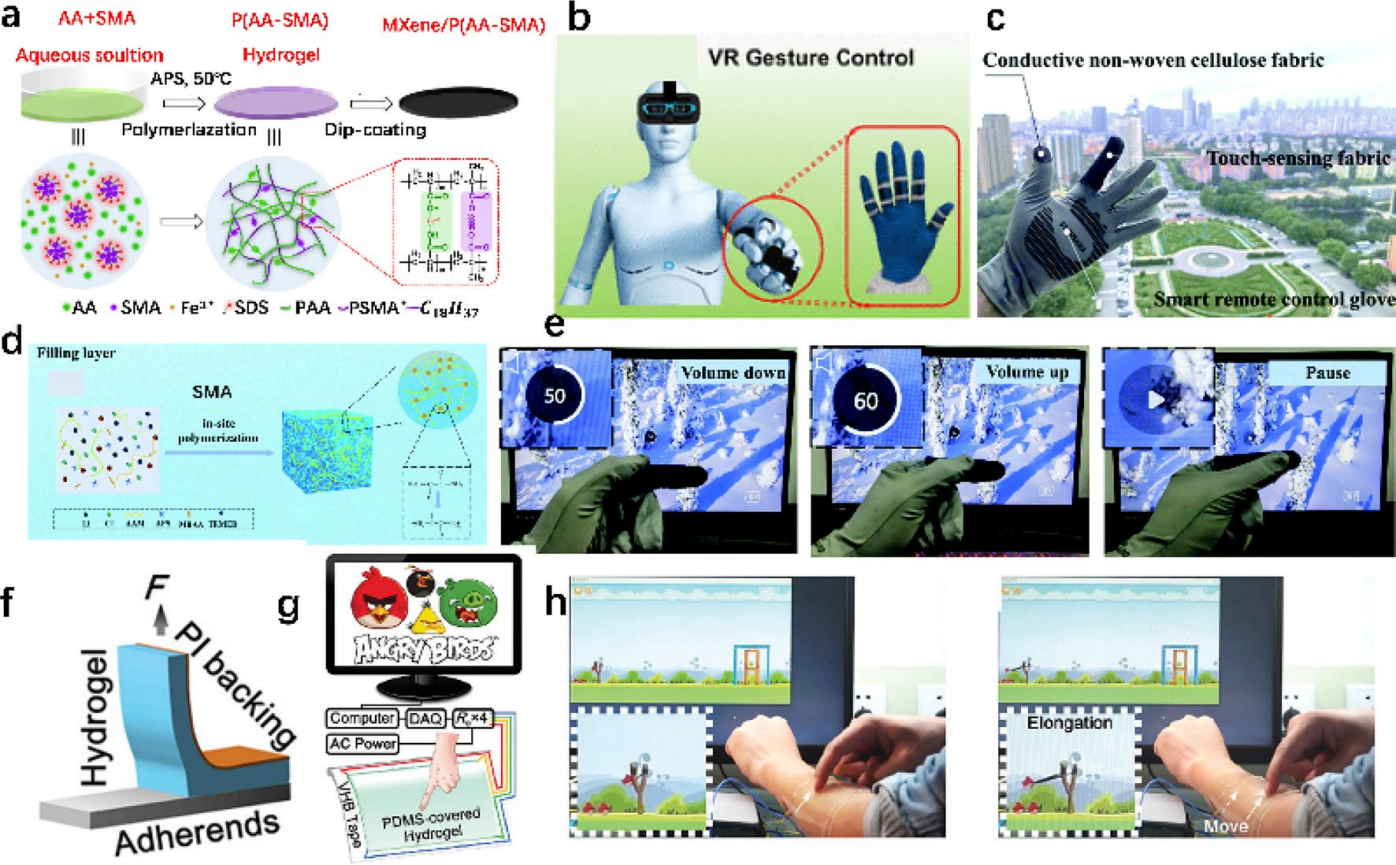
Applications of nanocomposite hydrogels in virtual reality and gaming interaction. **a**, **b** Ultra-stretchable, multi-repairable, and highly sensitive stress sensors based on dual-crosslinked MXene hydrogels, designed for precise gesture control in virtual reality applications [199]. **c**-**e** Tactile sensing fabrics encapsulated in hydrogels for human–computer interaction [200]. Copyright 2021, Royal Society of Chemistry. **f**–**h** Biomimetic self-healing human–computer interaction templates with pressure-sensitive adhesion [201]. Copyright 2020, Wiley

Gesture recognition and control technologies hold broad application potential in fields such as medical electronics, flexible displays, VR/AR, smart robotics, and gaming entertainment, providing new opportunities to technological advancement and industrial upgrading [202, 203]. To monitor subtle human movements and recognize different handwriting and gestures, Jie et al. [199] developed a dual-crosslinked poly(acrylic acid-stearoyl methacrylate)/MXene [P(AA-SMA)M] hydrogel with enhanced mechanical stretchability and self-healing properties. Figure 10a illustrates the preparation of the P(AA-SMA)M hydrogel film and the corresponding interactions within the hydrogel polymer network, including the copolymerization of AA and SMA, and the surface coating of MXene. The resulting sensor device can not only adhere to various body parts to monitor a wide range of physiological activities (e.g., limb movement, smiling, swallowing, wrist pulse, and sound vibrations) but also distinguish between different handwriting and gestures. Figure 10b shows a schematic diagram and actual photo of a skin sensor for VR gesture control, where the hydrogel sensors are attached to the five finger joints of the human hand. Combining multiple strain sensors into a specialized component can collect tactile signals, enabling virtual reality (VR) gesture control. An intelligent sensing platform was constructed to obtain command signals for virtual interface control through hand movement detection. This platform consists of five independent strain sensors installed at joints to detect the bending movements of each finger. It was observed that the hydrogel stress sensors accurately differentiate the response signals for each finger joint movement and record the corresponding resistance changes. The excellent capability to perceive different gestures makes the P(AA-SMA)M hydrogel strain sensors promising candidates for multifunctional sensor electronics and human–machine interfaces.

Flexible touch-sensitive devices have garnered significant attention in wearable electronics and human–machine interaction fields [204]. Intelligent touch-sensitive gloves exhibit tremendous application potential in wearable devices and HMI, offering convenient solutions for remote control and personalized command input while signaling a new trend of deep integration of smart clothing into daily life. To better facilitate remote command and HMI, Xu et al. [200] designed an intelligent touch-sensitive glove. In their study, they wrapped conductive Polyacrylamide-LiCl hydrogel with flexible nonwoven cellulose fabric to assemble an ultra-thin layered touch-sensitive fabric (1 mm). In this novel composite structure, the flexible nonwoven cellulose fabric serves as the outer layer, providing excellent mechanical properties and comfort. Figure 10c shows images and functions of the smart remote control glove, where the touch-sensitive area is divided into upper, lower, and space sections. Touching these sections in sequence causes a linear increase in the touch current (7.51 μA, 8.87 μA, 11.43 μA). Figure 10d illustrates the polymerization process of PAAM-LiCl hydrogel, which involves first depositing graphene onto the fabric using capillary deposition effects, then spraying a finishing agent to impart waterproof characteristics, and finally drying at 25 °C for 30 min. Figure 10e demonstrates various functions of the composite hydrogel for HMI, such as decreasing and increasing volume when touching the down and up buttons, respectively, and pausing video when touching the space button. The video resumes transmission after removing the glove. This tactile sensing fabric can be integrated into clothing to enable wearable HMI, showcasing significant potential in wearable interactive fields. In the context of VR/AR applications, this technology can enhance user experience by providing haptic feedback, enabling more immersive and interactive environments. By integrating such tactile sensing fabrics into VR/AR systems, users can physically interact with virtual objects or control virtual environments through gestures or touch, improving the realism and interactivity of these technologies.

Human–machine interaction (HMI) touchpads have a wide range of applications, including smart wearables, medical assistance, virtual reality games, and remote control, offering users unprecedented flexibility and convenience. To develop a single material system that integrates transparency, conductivity, stretchability, biocompatibility, self-healing, and self-adhesive properties, Gao et al. [201] developed a biomimetic self-healing HMI touchpad. This touchpad uses a polyzwitterionic-clay nanocomposite hydrogel as a transparent conductor, and exhibits pressure-sensitive adhesive properties on various substrates. Figure 10f shows a 90° peel-off schematic for the hydrogel adhered to the different materials, evaluating the adhesion performance of the hydrogel-substrate interface, indicating that the hydrogel serves as a pressure-sensitive adhesive on various solid surfaces. Figure 10g illustrates the wearable touchpad setup. VHB tape is used as an insulating spacer between the hydrogel and the body, with the hydrogel covered by PDMS adhered to the tape surface. Metal wires are then inserted into the four corners of the tape-hydrogel interface, connecting the hydrogel to a computer to create a wearable touchpad. Figure 10h demonstrates the touchpad worn on the arm while playing the popular video game Angry Birds. When a finger touches any part of the touchpad, the slingshot in the game is activated. By moving the finger on the touchpad, the slingshot with the bird is stretched and tightened. The scientific concepts and disruptive technology presented here open up possibilities for using polymer nanocomposite hydrogels as flexible HMI interfaces with self-healing properties.

### Robotic control

Nanomaterial composite hydrogel sensors exhibit unprecedented application potential in the field of robotic control, spearheading innovations in automation and intelligent technology [205–207]. From precise gesture imitation and dynamic environmental sensing to complex biological signal interaction and energy self-sufficiency, these sensors are progressively becoming indispensable core components in robotic control systems. Their unique material properties, such as high sensitivity, exceptional flexibility, excellent biocompatibility, and potential self-healing capabilities, provide robots with more intelligent, flexible, and adaptive control solutions [208, 209]. As technology continues to advance and materials science delves deeper, we anticipate that nanomaterial composite hydrogel sensors will catalyze numerous innovative applications across various dimensions of robotic control, propelling robotics technology towards higher levels of intelligence and autonomy [210, 211].

**Fig. 11:**
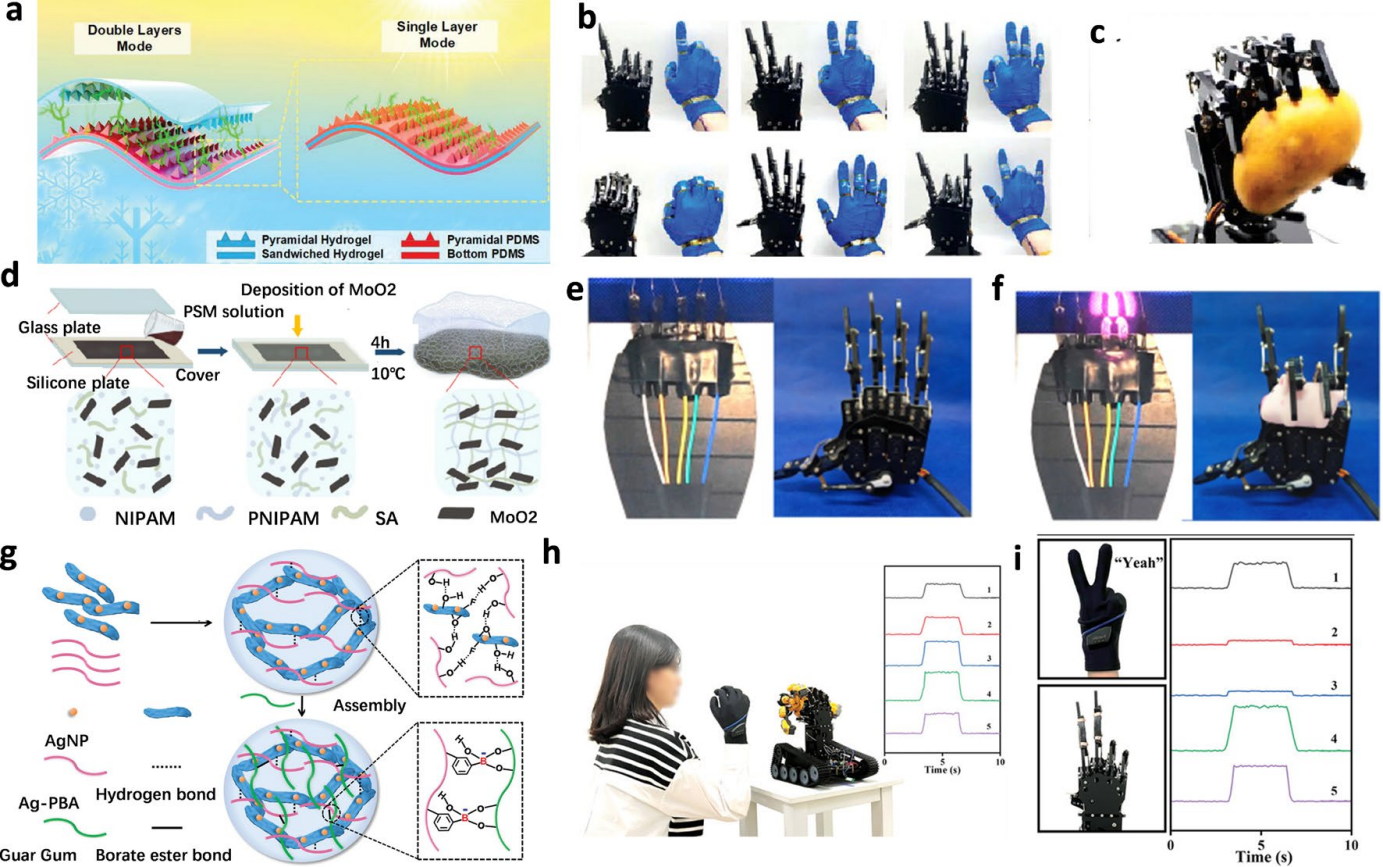
Applications of nanocomposite hydrogels in robotic control [212]. **a**-**c** Ultra-sensitive, deformable, and transparent triboelectric tactile sensors based on micro-pyramid-patterned hydrogels for interactive human–machine interfaces. Copyright 2022, Wiley. **d**-**f** Biomimetic multifunctional self-sensing and actuating gradient hydrogels for remote interaction in soft and hard robotics [213]. Copyright 2024, Springer Nature. **g**-**i** Flexible antibacterial MXene-based epidermal sensors that accelerate wound healing for intelligent wearable human–machine interaction [214]. Copyright 2022, Wiley

In the field of robotic control, where complex and harsh external environments are often encountered, the environmental tolerance of hydrogels is crucial. To enhance the environmental durability of TENG sensors for robotic applications, Chen et al. [212] fabricated a film-structured lithium bromide immersion treatment (LBIT) ion-based double network hydrogel and employed it in tactile sensors to achieve superior performance. Figure 11a illustrates the detailed structure, anti-dehydration, and anti-freezing properties of the highly deformable dual-electrode triboelectric hydrogel sensor (DE-THS) and single-electrode triboelectric hydrogel sensor (SE-THS). Based on the LBIT ion-type DN hydrogel film structure and different operating principles, two types of THS were proposed: the dual-electrode triboelectric hydrogel sensor (DE-THS) and the single-electrode triboelectric hydrogel sensor (SE-THS). Both DE-THS and SE-THS consist of a combination of PDMS and hydrogel films, forming a highly transparent and ultra-flexible film structure. Figure 11b demonstrates the gesture of a human hand and the corresponding movement response of a robotic hand. SE-THS can withstand various deformations, including stretching and twisting, exhibiting excellent flexibility. Five SE-THS sensors are attached to the finger joints of a human hand to monitor bending movements, which can be used to control a robotic hand. Figure 11c shows a robotic hand grasping a mango, demonstrating the exceptional sensitivity of SE-THS in detecting finger joint movements. The SE-THS sensors attached to multiple fingertips show no signal interference or noticeable signal delays. Integrated with human–machine interface systems, SE-THS holds great promise for future robotic surgical systems and manufacturing applications under extreme temperature conditions.

Hydrogels have wide applications and great potential in robotic systems, particularly in achieving remote interaction between soft and rigid robots. Liu et al. [213] developed a bioinspired multifunctional self-sensing actuator gradient hydrogel. Figure 11d illustrates the synthesis, polymer dispersion, and gradient network structure of PSM hydrogel. A mixture of Sodium Alginate(SA), MoO₂, and N-Isopropylacrylamide(NIPAM)monomers was poured into a mold, then covered with a glass plate. During polymerization, MoO₂ nanosheets rapidly settled to the bottom of the mixed solution due to gravity. The hydrophilic NIPAM monomers were more attracted to the top, driven by the hydrophobic nature of the MoO₂ nanosheets, resulting in a rich PNIPAM phase at the top. Consequently, the hydrogel achieved a gradient structure along the direction of gravity. This hydrogel strain sensor demonstrated excellent sensitivity, including a gauge factor (GF) of 3.94, high stretchability (600%), fast response time (140 ms), and reproducible stability. Figure 11e and f show the self-sensing hydrogel actuator controlling a Bluetooth-interactive robotic hand to grab a small toy pig before and after Near Infrared (NIR) stimulation. By integrating actuation and sensing capabilities, a self-sensing biomimetic tongue was fabricated and combined with the Internet of Things (IoT) to quantify the movement trajectory of the soft actuator, thereby enabling a remote control system for NIR-stimulated response, with the self-sensing hydrogel actuator controlling a Bluetooth-interactive robotic hand. This work presents a novel and efficient method for fabricating high-performance multifunctional self-sensing actuator gradient hydrogels, paving the way for the development of next-generation intelligent interactive haptic soft materials.

Composite hydrogel-based epidermal sensors hold great potential for wide applications in smart healthcare and assistive technologies. To sensitively monitor both large human movements and subtle electrophysiological signals, thus enabling human–machine interaction control, Li et al. [214] developed an epidermal sensor made from MXene hydrogel. This sensor, with enhanced mechanical properties, excellent conductivity, and impressive sensing performance, is suitable for personal medical monitoring and disease prediction. Figure 11g illustrates the fabrication of the MXene hydrogel. By incorporating a network of antibacterial silver nanoparticle-coated MXene (AgNPs/MXene) nanosheets into a polymer matrix of guar gum (GG) and phenylboronic acid-grafted sodium alginate (Alg-PBA), a simple method was developed to create a self-healing, injectable, and antibacterial MXene hydrogel for wearable human–machine interaction and high-performance healthcare monitoring. In Fig. 11h, the flexible epidermal sensor is attached to the fingers of a robotic hand. When a volunteer wears a wireless motion-sensing glove, the robotic hand can be wirelessly controlled by the user’s hand gestures. The sensor allows the robotic hand to sense and replicate the volunteer’s gestures. Figure 11i shows a volunteer bending their fingers to make a “Yes” gesture. The wireless motion-sensing glove, paired with the flexible epidermal sensor, enables the robotic hand to perceive real-time gestures, such as a “Yeah” hand sign, and execute corresponding grip movements. Thus, the MXene hydrogel-based epidermal sensor shows significant promise in applications such as sign language translation, multifunctional prosthetics, and intelligent human–machine interactions.

In summary, the performance differences of nanocomposite hydrogels in similar application scenarios are mainly in terms of conductivity, mechanical properties, sensitivity and stability. Carbon nanomaterial composite hydrogel is excellent in improving the sensitivity and mechanical strength of strain sensors, and is suitable for monitoring human movement; metal nanomaterial composite hydrogel has advantages in fast response as well as high sensitivity, and is suitable for biosensors and touch panels, but there is the problem of metal particles aggregation, which affects the homogeneity and long-term stability;. In contrast, MXene composite hydrogel demonstrates good mechanical flexibility and electrical conductivity, which is suitable for wearable devices and flexible electronic devices. Still, it faces challenges in mechanical strength due to its weak interaction with polymer networks [215, 216]. The performance comparisons for different applications of hydrogel-based sensors are summarized in Table 2.

**Table 2:** Performance comparison of hydrogel-based sensors in various application domains

Application Domain	Key Material/Technology	Performance Features	References
Personal Electronic Devices	Carbon nanotubes (CNTs), graphene-based hydrogels	High sensitivity, self-healing, flexible strain sensing	[61, 63, 64]
Wearable Health Monitoring	MXene nanomaterial-based hydrogels	Real-time signal monitoring, high conductivity, biocompatibility	[20, 102, 103]
VR/AR Interaction	MXene hydrogels, dual-crosslinked networks	Gesture recognition, rapid signal response	[199, 200]
Robotic Control	AgNW, liquid metal-based hydrogels	Precise motion detection, strain-resistance	[79, 80, 214]
Electrophysiological Monitoring	PEDOT hydrogel electrodes	High SNR, skin adhesion, scalable fabrication	[164, 166]

## Conclusion and future perspectives

In summary, composite hydrogels based on nanomaterials have demonstrated immense potential in the fields of flexible, wearable, and highly sensitive human–machine interaction sensors. By incorporating carbon nanomaterials, metallic nanomaterials, and MXene into hydrogels, these nanocomposites have significantly improved mechanical properties, conductivity, and flexibility, making them ideal candidates for various sensing applications. Each type of nanomaterial offers unique advantages: carbon nanomaterials provide exceptional conductivity and mechanical strength, metallic nanomaterials exhibit excellent electrical performance and flexibility, while MXene materials display outstanding conductivity, hydrophilicity, and electrochemical stability. The sensing mechanisms of these hydrogels are diverse, including triboelectric nanogenerator principles, stress-resistance response mechanisms, and electrophysiological signal acquisition mechanisms. These mechanisms enable sensors to accurately detect various external stimuli, such as mechanical deformations and physiological signals, paving the way for applications in personal electronics control, VR/AR gaming interactions, and robotic systems.

However, despite the remarkable progress that has been achieved in nanocomposite hydrogel sensors, several challenges remain. Firstly, the long-term durability of composite hydrogels needs further enhancement to ensure reliability in practical applications. Future research could focus on optimizing chemical crosslinking methods to enhance mechanical strength and reduce degradation in hydrogels. Secondly, scalable manufacturing processes are desirable to meet the growing demands for flexible electronics in consumer and industrial markets. Future research should aim to develop cost-effective, scalable manufacturing methods for hydrogels, optimizing techniques like spray coating, 3D printing, and slot-die coating to reduce costs and improve efficiency. In addition, the environmental adaptability of hydrogels is relatively poor, and their performance may fluctuate under different humidity and temperature conditions, affecting their stability and accuracy. Future research could focus on developing hydrogels with better environmental adaptability, such as by introducing dynamic responsive functions that allow them to self-adjust based on changes in humidity and temperature. Lastly, integrating artificial intelligence algorithms with real-time data analysis into these sensors can further expand their application scope, making human–machine interaction systems more intuitive and efficient. In the future, interdisciplinary research in materials science, electrical engineering, and biomedicine will be crucial for advancing nanomaterial-based composite hydrogel sensors. With continued optimization, these advanced materials are promising to play a pivotal role in shaping the next generation of human–machine interactions, driving innovation in healthcare, robotics, and consumer electronics.

## Data Availability

No datasets were generated or analysed during the current study.
